# Identification of novel HNF1B mRNA splicing variants and their qualitative and semi-quantitative profile in selected healthy and tumour tissues

**DOI:** 10.1038/s41598-020-63733-x

**Published:** 2020-04-24

**Authors:** Jan Hojny, Michaela Bartu, Eva Krkavcova, Kristyna Nemejcova, Jan Sevcik, David Cibula, Vladimir Fryba, Lenka Plincelnerova, Pavel Dundr, Ivana Struzinska

**Affiliations:** 1 Institute of Pathology, First Faculty of Medicine, Charles University and General University Hospital in Prague, Prague, 12808 Czech Republic; 2 Institute of Biochemistry and Experimental Oncology, First Faculty of Medicine, Charles University, Prague, 12853 Czech Republic; 3 Gynecological Oncology Center, Department of Obstetrics and Gynecology, First Faculty of Medicine, Charles University and General University Hospital in Prague, Prague, 12851 Czech Republic; 4 1st Department of Surgery - Department of Abdominal, Thoracic Surgery and Traumatology, First Faculty of Medicine, Charles University and General University Hospital in Prague, Prague, 12808 Czech Republic; 5 Department of Urology, First Faculty of Medicine, Charles University and General University Hospital in Prague, Prague, 12808 Czech Republic

**Keywords:** Transcription, Cancer, Biomarkers

## Abstract

Hepatocyte nuclear factor-1-beta (HNF1B) is a transcription factor crucial for the development of several tissues, and a promising biomarker of certain solid tumours. Thus far, two HNF1B alternative splicing variants (ASVs) have been described, however, the complete spectrum, prevalence and role of HNF1B ASVs in tumorigenesis are unclear. Considering the equivocal data about HNF1B ASVs and expression presented in literature, our aim was to characterize the spectrum of HNF1B mRNA splicing variants across different tissues. Here, we characterize HNF1B ASVs with high sensitivity in carcinomas of the uterine corpus, large intestine, kidney, pancreas, and prostate, with selected paired healthy tissues, using the previously described multiplex PCR and NGS approach. We identified 45 ASVs, of which 43 were novel. The spectrum and relative quantity of expressed ASVs mRNA differed among the analysed tissue types. Two known (3p, Δ7_8) and two novel (Δ7, Δ8) ASVs with unknown biological functions were detected in all the analysed tissues in a higher proportion. Our study reveals the wide spectrum of HNF1B ASVs in selected tissues. Characterization of the HNF1B ASVs is an important prerequisite for further expression studies to delineate the HNF1B splicing pattern, potential ASVs functional impact, and eventual refinement of HNF1B’s biomarker role.

## Introduction

**Figure 1:**
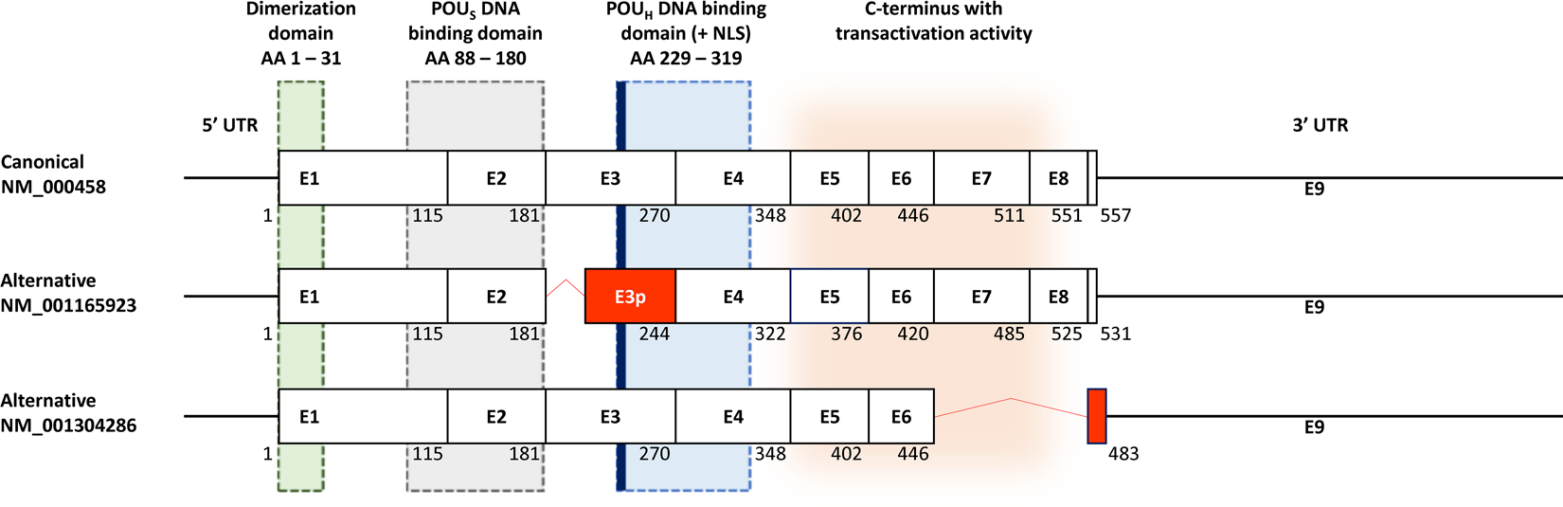
Scheme of the currently known canonical and alternative HNF1B transcripts (according to the RefSeq database, accessed January 10, 2020). Alternative transcript NM_001165923 lacks 26 AA (78 bp) at the 5′ end of exon 3 (red box; named exon 3p). Alternative transcript NM_001304286 lacks the entire exons 7 and 8 which results in reading frame shift of exon 9 coding part (red box) and use of alternative STOP codon (92 bp after original STOP codon). The lengths of the exons are proportional. The white boxes illustrate the unaffected coding exons. Corresponding amino acid (AA) numbers for each isoform are indicated below the exon boxes. The green, grey, blue and orange areas illustrate the coding areas for functional domains across the HNF1B transcripts. UTR – untranslated region. NLS – nuclear localization signal (thick blue line). POU_S_ – POU specific domain. POU_H_ – POU homeodomain. The scheme was adopted^2^ and modified.

Hepatocyte nuclear factor 1 beta (HNF1B, also known as Transcription Factor-2, TCF2), belongs to a family of transcription factors which are crucial for the regulation of the development of various tissues and organs during embryogenesis. Although originally described in the liver, HNF1B also plays an important role in the development and differentiation of the kidney, pancreas, reproductive tract, and biliary system^1–3^. The *HNF1B* gene comprises 9 exons and codes for a protein with 3 important functional domains: the N-terminal dimerization domain, the DNA-binding domain (consisting of the Pit1/Oct-1/Unc-86-POU-homeodomain and a POU-specific domain), and the C-terminal transactivation domain (Fig. 1)^4^. Apart from its role during organogenesis in the embryonic stage, in adults HNF1B acts as a classic transcription activator of the expression of multiple genes implicated in cell cycle regulation, apoptosis, glucose metabolism^5–7^, and as a regulator of the expression of genes associated with stem or progenitor cells^3^. HNF1B is expressed mainly in tubule-forming epithelial tissues, such as kidney or pancreatic exocrine duct tubules, and also in the gall bladder, colon, duodenum, intestine, lung, stomach, urinary bladder, liver, endometrium, prostate, testis, and appendix^3,8^.

Besides the known developmental disorders and syndromes associated with inactivating mutations in the *HNF1B* gene^9,10^, there is evidence that HNF1B expression is associated with the tumorigenesis of several types of solid tumours, especially in the subset of clear cell carcinomas of the ovary (OCCC)^6,7^ and renal cell carcinomas (RCC) of the kidney^11^. While the higher HNF1B expression in OCCC corelates with a higher cancer risk^6,7^, on the contrary in RCC it is the lower HNF1B levels which are associated with tumour progression and poor prognosis^11^. Moreover, its role and expression levels in the development of tumours of the liver, gastrointestinal tract, pancreas, prostate, colorectal carcinoma, as well as endometrial tumours and non-tumour lesions, is also being discussed with ambiguous conclusions^2,3,12–14^.

It is now commonly accepted that alternative splicing or its deregulation may play an important role in the tumorigenesis of certain cancer types^15,16^. Textbook examples which support the importance of alternative splicing and its influence on protein functions are certain BRCA1 alternative splicing variants (ASVs), which lead to translation into protein isoforms lacking important conservative domains. As a result, proteins with a low functional level are formed and negatively influence/regulate the BRCA1 biological function^17,18^. The knowledge of the expression levels of HNF1B mRNA variants or protein isoforms is crucial for the precise interpretation of HNF1B as a prognostic marker in a wide spectrum of expression studies. However, the current results are unclear and sometimes contradictory.

According to the current NCBI and Ensemble databases (accessed January 10, 2020), three fully characterized HNF1B transcripts and their protein products are known (the full-length NM_000458 and two alternatively spliced variants NM_001165923 and NM_001304286, Fig. 1). Interestingly, the second alternatively spliced variant lacks two exons in a coding area for the transactivation domain, which may essentially influence the functional effect of this isoform (as mentioned above). To the best of our knowledge, qualitative and quantitative profiles of these variants in different lesions, as well as their functional potential, have not been investigated yet.

Therefore, the aim of our study was to precisely describe the spectrum of HNF1B ASVs in different types of tumour and corresponding healthy tissues. This qualitative and semi-quantitative characterization of HNF1B ASVs pattern in selected tissues is an inevitable step for the further analysis of HNF1B expression, and therefore for a precise interpretation of HNF1B as a prognostic biomarker.

## Results

### Analysis of selected tissue pools confirmed 2 known ASVs and revealed 43 novel HNF1B ASVs

The combined mPCR (multiplex PCR)/NGS (next generation sequencing) approach revealed a total of 45 HNF1B splicing variants (events) across 11 pools of analysed tissue types (Table 1), including 43 novel ASVs. Eleven variants were expressed ubiquitously across all the analysed tissue pools (3p; Δ5; Δ6_8; Δ7,6q,8p; Δ7; Δ7_8; Δ8; ▼157bp_i4; ▼91bp_i4; ▼128bp_i4; ▼148bp_i5). Four of these variants, two previously reported (3p, Δ7_8) and two novel (Δ7 and Δ8), were expressed with high read counts (>1000) in a majority of the analysed tissue pools, and so we marked them as “predominant” variants. Two other ASVs were detected with read counts >1000 in more than 4 pools (Δ5_8 was detected in the total of 10 pools and Δ6_8 in all 11 pools, Table 1), and therefore we indicated them as “predominant candidate” variants. The relative quantity of the other 39 variants was evaluated, by our semi-quantitative approach, as rather lower (>1000 normalized reads in the majority of the analysed tissue pools).

**Table 1:** List of the identified HNF1B ASVs in eleven pools of different tissue types.

Variant name	HGVS description	Functional annotation	Splicing biotype	Endometrioid endometrial carcinoma	Colorectal carcinoma	Healthy colon	Kidney carcinoma	Healthy kidney	Kidney oncocy-toma	Pancreatic carcinoma	Healthy pancreas	Prostate carcinoma	Paired healthy tissue	Prostate hyper-plasia	No. of pools with ASV	Novel (0), known (1)
Δ2	c.345_544 del200	FS	CΔ	255	0	1 454	199	754	259	322	556	0	0	444	8	0
Δ2,3p	c.345_622 del278	FS	CΔ + SDSΔ	0	143	0	127	436	163	64	0	0	136	385	7	0
Δ2_4	c.345_1045 del701	FS	mCΔ	68	0	57	468	166	216	143	91	0	0	0	7	0
Δ2_5	c.345_1206 del862	FS	mCΔ	0	0	0	34	14	0	0	0	0	0	0	2	0
Δ2_6	c.345_1339 del995	FS	mCΔ	0	0	0	770	0	0	0	0	0	0	1 719	3	0
Δ2_7,8p	c.345_1584 del1204	FS	mCΔ + SASΔ	0	0	0	0	117	0	0	0	0	0	0	1	0
Δ2_8	c.345_1653 del1309	FS	mCΔ	0	0	226	951	844	276	433	275	446	0	0	7	0
**3p**	**c.545_622 del78**	**IF**	**SASΔ**	**89 082**	**166 213**	**185 492**	**178 626**	**179 688**	**130 503**	**182 721**	**163 105**	**227 604**	**199 210**	**273 908**	**11**	**1**
Δ3_4	c.545_1045 del501	IF	mCΔ	19	138	89	199	237	74	303	221	0	0	0	8	0
Δ3_5	c.545_1206 del662	FS	mCΔ	0	0	0	0	0	5	0	0	0	0	0	1	0
Δ3_5,6p	c.545_1328 del784	FS	mCΔ + SASΔ	0	0	0	132	0	0	0	0	0	0	0	1	0
Δ3_8	c.545_1653 del1109	FS*	mCΔ	0	0	0	0	139	0	0	0	0	0	0	1	0
Δ4_5,3q,6p	c.796_1222 del427	FS	mCΔ + SDSΔ + SASΔ	0	0	0	0	22	0	0	0	0	0	0	1	0
Δ4,3q	c.799_1045 del247	FS	CΔ + SDSΔ	0	0	0	0	0	0	16	0	0	0	0	1	0
Δ4_6,3q,7p	c.804_1359 del556	FS	mCΔ + SDSΔ + SASΔ	8	0	0	0	0	0	0	0	0	0	0	1	0
Δ4	c.810_1045 del236	FS	CΔ	0	0	39	41	54	0	292	116	0	0	0	5	0
Δ5_7,4q,8p	c.1039_1548 del510	IF	mCΔ + SDSΔ + SASΔ	0	0	59	0	0	0	0	0	0	0	0	1	0
Δ5_6,4q,7p	c.1041_1520 del480	FS	mCΔ + SDSΔ + SASΔ	0	0	519	0	0	0	0	0	0	0	0	1	0
Δ5	c.1046_1206 del161	FS	CΔ	135	77	271	674	365	187	884	411	634	694	336	11	0
Δ5,6p	c.1046_1222 del177	IF	CΔ + SASΔ	0	7	0	18	0	46	0	0	0	0	4	4	0
Δ5,6p	c.1046_1225 del180	IF	CΔ + SASΔ	0	0	0	8	0	0	0	0	0	0	0	2	0
Δ5_6	c.1046_1339 del294	IF	mCΔ	0	0	0	158	621	0	0	0	0	0	0	2	0
Δ5_7	c.1046_1534 del489	IF	mCΔ	0	0	0	594	0	0	0	0	0	0	0	1	0
**Δ5_8**	**c.1046_1653 del608**	IF*	**mCΔ**	**113**	**208**	**866**	**2 739**	**2 324**	**2 145**	**786**	**927**	**0**	**407**	**1 064**	**10**	**0**
Δ6_7,5q	c.1199_1534 del336	IF	mCΔ + SDSΔ	0	0	229	0	5	0	26	0	0	0	0	3	0
Δ6	c.1207_1339 del133	FS	CΔ	191	63	101	269	155	190	240	310	540	0	0	9	0
Δ6_7	c.1207_1534 del328	FS	mCΔ	0	0	0	545	152	411	263	108	0	0	0	5	0
**Variant name**																
Δ6_8	c.1207_1653 del477	IF	mCΔ	566	599	637	3 527	3 155	1 708	899	2 065	647	354	2 629	11	0
Δ7,6q,8p	c.1336_1542 del207	IF	CΔ + SDSΔ + SASΔ	68	5	12	8	24	60	33	25	27	46	17	11	0
**Δ7**	**c.1340_1534 del195**	**IF**	**CΔ**	**13 053**	**25 843**	**25 407**	**31 199**	**29 225**	**27 652**	**34 884**	**29 461**	**31 266**	**25 085**	**38 698**	**11**	**0**
**Δ7_8**	**c.1340_1653 del314**	IF*	**mCΔ**	**8 541**	**39 834**	**38 134**	**69 676**	**76 681**	**84 948**	**72 312**	**45 479**	**24 189**	**26 958**	**45 290**	**11**	**1**
**Δ8**	**c.1535_1653 del119**	IF*	**CΔ**	**1 399**	**4 506**	**5 749**	**6 095**	**6 421**	**5 017**	**3 441**	**3 690**	**871**	**1 713**	**1 098**	**11**	**0**
▼106bp_i2	c.545-899_549-793 ins106	FS	C▼	64	0	0	0	0	17	0	5	0	0	0	3	0
▼153bp_i4	c.1046-10281_1046-10129 ins153	IF, PTC	C▼	7	7	6	7	7	0	7	19	6	2	0	9	0
▼157bp_i4	c.1046-10281_1046-10125 ins157	FS	C▼	30	26	16	29	17	4	26	24	9	11	6	11	0
▼91 bp_i4	c.1046-10219_1046-10129 ins91	FS	C▼	0	0	0	4	0	0	0	0	0	0	0	1	0
▼94 bp_i4	c.1046-10219_1046-10125 ins94	FS	C▼	0	0	4	0	0	0	2	0	0	0	0	2	0
▼91 bp_i4	c.1046-1421_1046-1331 ins91	FS	C▼	928	216	204	597	462	178	312	460	236	399	251	11	0
▼128bp_i4	c.1046-765_1046-638 ins128	FS	C▼	98	54	44	101	72	24	44	65	32	86	35	11	0
▼99 bp_i4	c.1046-1421_1046-1331 ins91 + c.1046-8_1046-1 ins8	IF	C▼ + SAS▼	24	0	0	0	0	0	2	0	14	0	0	3	0
▼79 bp_i5	c.1206 + 1417_1206 + 1495 ins79	FS	C▼	0	0	14	0	0	2	0	0	0	0	5	3	0
▼148bp_i5	c.1206 + 1417_1206 + 1564 ins148	FS	C▼	368	75	41	231	162	38	108	185	82	107	15	11	0
▼195bp_i6, Δ7_8	c.1339 + 521_1339 + 715 ins195 + c.1340_1653 del314	FS	C▼ + mCΔ	0	0	0	52	0	0	0	0	0	0	0	1	0
▼92 bp_i6	c.1339 + 1620_1339 + 1711 ins92	FS	C▼	0	0	0	0	0	0	8	0	0	0	0	1	0
▼169bp_i6	c.1340-1781_1340-1613 ins169	FS	C▼	34	40	22	109	10	3	58	39	4	5	0	10	0
ASV reads in pool together				115 049	238 054	259 694	298 184	302 328	254 125	298 628	247 635	286 606	255 213	365 903		
ASV reads in pool together (% of normalized million)				11,5	23,8	26,0	29,8	30,2	25,4	29,9	24,8	28,7	25,5	36,6		
No. of ASVs in pool				21	18	25	31	28	24	27	22	16	15	17		

Cells with stated normalized sequencing coverage higher than 1000 are underlined. The “predominant” and the “predominant candidate” variants are in **bold**. The list includes the variant’s name, presumed HGVS nomenclature, presumed functional annotation (FS = frameshift; IF – in frame; FS* - frameshift in the last exon 9 with alternative STOP codon), splicing event biotype (Δ = deletion; ▼ = insertion; C = cassette/exon; mC = multicasette/multiexon; SDS = splice donor site shift; SAS = splice acceptor site shift).

Considering the potential impact on the mRNA sequence, thirteen of the detected ASVs maintain the original open reading frame, while 32 ASVs cause frameshift including three novel variants with exon eight deletion (Δ3_8; Δ5_8 and Δ8). Interestingly, based on the sequencing data, these three ASVs maintain the same open reading frame with frameshift in the last exon (exon 9) as was described previously for variant Δ7-8 (NM_001304286; Fig. 1).

The existence of the predominant ASV Δ8 in the full-length HNF1B alternative transcript form (HNF1BΔ8; including canonical exon 3 variant) was proven by the sequencing analysis of the cloned transcript, isolated from the non-tumour ovary tissue during the method optimization steps (as described in the Materials and Methods section).

### Several ASVs showed tissue specific or tumour specific expression levels

The evaluation of ASVs with a reads count >1000 in the analysed tissue pools showed tissue specific expression in several cases. The ASV lacking the first 78 bp of canonical exon 3, the 3p variant (NM_001165923; Fig. 1) shows a relatively low normalized expression in the EEC pool (endometrial endometroid carcinoma; 89082 reads), in contrast to the prostate lesion pools (carcinoma – 227604 reads and hyperplasia – 273908 reads) where the expression is much higher. Another highly expressed predominant ASV Δ7 also showed major expression differences between the EEC pool (13053 reads were detected) and all the other pools, in which the expression was almost two to three times higher (25407–38698 reads). Interestingly, the prostate hyperplasia pool was the only pool where ASV Δ2_6 was detected in higher read counts (1719 reads) in contrast to the other tissue pools.

The comparison of the expression between the paired normal and cancer tissues also showed some differences. The expression of the predominant ASV Δ7_8 was 59% higher in the pancreatic carcinoma pool (72312 reads), than in the pool of healthy pancreatic tissue (45479 reads). Furthermore, minor read count differences were also detected for the following ASVs: Δ2 in the large intestine pools (1454 reads in the healthy large intestine tissue, with no expression in the paired tumour tissue), Δ6_8 in the pancreas pools (2065 reads in the healthy pancreas versus 899 reads in the pancreatic cancer tissue). The last minor Δ8 read number difference was observed in the prostate carcinoma pool (871 reads), and the healthy tissue pool, which is represented by seminal vesicles tissue (1713 reads).

### Spectrum and total portion of detected ASVs varies among analysed tissue pools

Out of the total of 45 ASVs detected in all the analysed tissue pools together, the numbers of ASVs in individual pools were lower and significantly different. In the prostate-related pools, 15 (healthy tissue - seminal vesicles), 16 (paired tumour), and 17 (hyperplasia) ASVs were observed in contrast to the broadest spectrum of detected ASVs in the kidney paired pools where 31 (kidney carcinoma), and 28 (healthy kidney) ASVs were detected (Table 1). The comparison of tumour and paired healthy tissues revealed an interesting qualitative difference between the colorectal carcinoma pool (only 18 variants) and the corresponding healthy colon pool (25 variants, including Δ2 variant with 1454 normalized reads), while the quantitative differences between all of the identified ASVs together in these pools were minimal (238 054 normalized reads in the carcinoma vs. 259 694 normalized reads in the healthy tissue pool; Table 1).

The total number of normalized ASV reads together in each tissue pool revealed a particularly high portion of the expressed ASVs in the prostate hyperplasia pool (365903 reads of all ASVs; 36.6%), when compared to the lower portion detected in the EEC pool (115049 reads of all ASVs; 11.5%). The remaining tissue pools were close to the median value calculated from all the tissue pools (273150 reads of all ASVs = 27.3% of all reads; range 23.8–30.2%; Table 1).

All the tissue pool specific ASVs (Δ2_7,8p; Δ3_5; Δ3_5,6p; Δ3_8; Δ4_5,3q,6p; Δ4,3q; Δ4_6,3q,7p; Δ5_7,4q,8p; Δ5_6,4q,7p; Δ5_7; ▼91 bp_i4 (exonization of 91bp in intron 4); ▼195bp_i6,Δ7_8 and ▼92bp_i6) were detected in rather low portions (<1000 reads; Table 1).

## Discussion

The role of *HNF1B* in tumorigenesis has not yet been fully clarified. The results of recent studies suggest that HNF1B may act as either a tumour suppressor or an oncogene, depending on the type of tissue and tumour^2^. There are studies which define HNF1B as a pro-differentiation factor with a potent tumour-suppressive activity in healthy tissues^6,7,19^, while other studies point to its role as an oncogene in tissue-derived cancer cells which have undergone malignant transformation, inducing a cancerous phenotype and activating the formation of invasive phenotypes through epithelial-mesenchymal transition^11,14^. One of the possible explanations for this ambivalent function of HNF1B could be a tumour-specific expression of HNF1B ASVs, or a dysregulation in the splicing pattern resulting in the expression of divergent protein isoforms.

The detection of a total of 45 variants proved that HNF1B splicing pattern is considerably complex. We identified the expression of four predominant variants (3p, Δ7, Δ7_8 and Δ8) detected in all the analysed tissue pools in a higher portion, and two predominant candidate variants (Δ5_8 and Δ6_8) detected in a majority of the pools (10/11 and 11/11, respectively). Analysis of individual tissue pools revealed qualitative (Δ2 in colorectum) and quantitative (Δ7_8 in pancreas) differences between the healthy and tumour tissues, and several low-expressed tissue specific ASVs. A majority of the detected variants showed a low expression pattern (<1000 normalized read counts) which suggests a rather low physiological impact of these variants. Moreover, according to the literature the estimation of the splicing error rate is relatively high (0.7%)^20^. Considering this estimation, detected variants with low normalized read counts, especially in single or low numbers of analysed pools, could be a consequence of splicing errors.

To our knowledge, only two studies have analysed the expression of mRNA HNF1B transcripts to date. In the first study^21^, mRNA expression of wt HNF1B variant (named HNF1B(B)), HNF1B 3p variant (named HNF1B(A)) and variant “HNF1B(C)” (variant with transcription alternatively stopped after exon 4) were analysed in selected human tissues (pancreas tissues, liver and kidney). Interestingly, the reported mRNA expression of ASV 3p was higher in pancreatic islet tissue and balanced in kidney tissue, in comparison to wt HNF1B, which proves the presumed predominant expression of the 3p transcript variant.

In another study^22^, the same authors described changes in the expression of the HNF1B 3p variant while comparing 39 non-malignant benign prostatic hyperplasia samples and 21 prostate adenocarcinomas. They concluded that there is only a minor difference in the expression of 3p ASV in the analysed tissues, which is comparable to our findings. The relative expression of the 3p variant in the discussed study is 1.28x higher in the hyperplasia tissues (1.2) when compared to tumour tissues (0.94), while our data shows a 1.20x higher expression in the hyperplasia pool (273908 normalized reads) compared to the tumour tissue pool (227604 normalized reads). This result supports the semi-quantitative character of the methodical approach used, especially in the paired tissues where the compared samples were obtained from the same individuals.

In comparison with the RNA-Seq GTEx Portal database (https://gtexportal.org/; focused on mRNA expression in human tissues; accessed January 10, 2020), our methodical approach shows significantly higher sensitivity^23^. Based on the exon usage analysis, the data presented in GTEx concerning HNF1B suggests and supports the existence and predominant expression of 3p, Δ7_8 and Δ8 variants. Due to the low coverage of HNF1B transcripts in this database, other ASVs are presumably under the detection limit, and thus ASVs detection capability of used GTEx algorithms is low for HNF1B. For the kidney, the tissue with the highest HNF1B expression, it shows that the median read count per base reaches only 6.81 for the best covered last exon 9; in other tissues it is under 1.2, usually in a range of 0.1–0.5.

Our data shows that the overall splicing pattern differs among several tissue pools. A lower complexity of ASVs, but the similar total number of ASV reads, was detected in the colorectal carcinoma pool (18 detected variants; 238 054 normalized reads), which is in contrast to the paired healthy colon samples (25 detected variants; 259 694 normalized reads). On the contrary, significant deviations in the total ASVs reads were observed in the prostate hyperplasia pool (365 903 normalized reads; 36.6%; 17 detected ASVs) and the EEC pool (only 115049 of all normalized reads belonged to ASVs; 11.5%; 21 selected ASVs) which suggests that the splicing pattern of HNF1B could be disease-specific according to the quality (paired colorectal pools) or quantity (EEC and prostate hyperplasia pools) in the analysed tissues.

**Figure 2:**
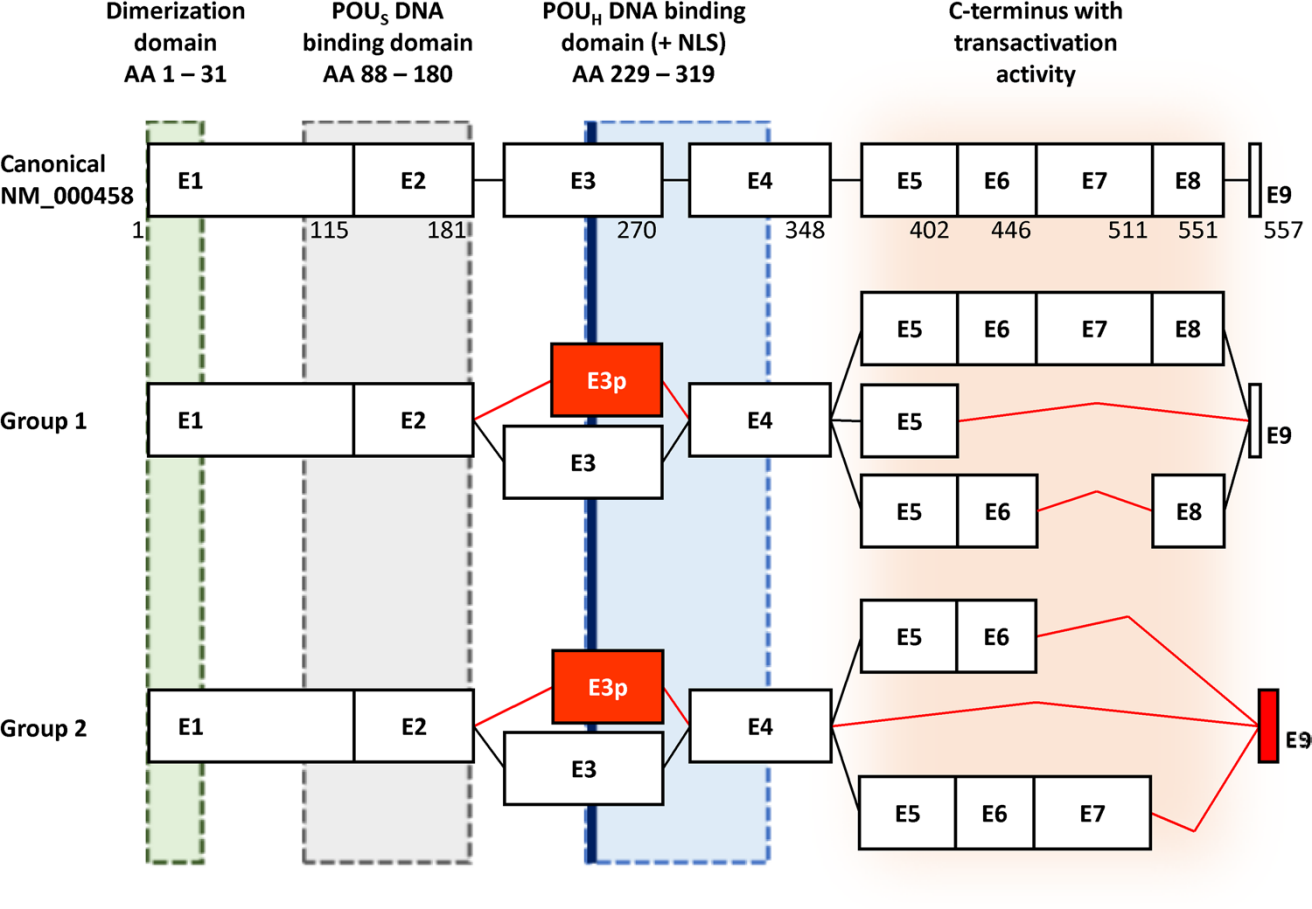
Scheme of the proposed HNF1B splicing pattern HNF1B alternative splicing variants can be divided into two main groups according the maintenance of original reading frame. ASVs in the first group maintain original reading frame across the whole transcript, including last exon 9, while ASVs in second group cause exon 9 frameshift (red box) which results in the use of alternative STOP codon (92 bp after original STOP codon). We propose, that all ASVs in each group will be present in both forms of exon 3, canonical or alternatively spliced (e3p; red box). Black lines connecting exon boxes represents canonical transcript, while red lines represent alternative splicing event. The lengths of the exons are proportional. The white boxes illustrate the unaffected coding exons. Corresponding amino acid (AA) numbers are indicated below the exon boxes of canonical isoform. The green, grey, blue and orange areas illustrate the coding areas for functional domains across the HNF1B transcripts. NLS – nuclear localization signal (thick blue line). POU_S_ – POU specific domain. POU_H_ – POU homeodomain. The scheme was adopted^2^ and modified.

Although our presented results, based on mPCR and NGS methods, are unable to reveal exact combination of individual splicing variants / events within individual HNF1B transcripts, we can propose HNF1B splicing pattern composed of these most occurred “predominant” and “predominant candidate” variants. These variants offer only a limited number of combinations; variants localized in the 3′ end of the transcript (C-terminus of protein) are mutually exclusive and may be combined only with the 3p or canonical exon 3 variant (Fig. 2). The exon 3p is probably the most expressed ASV, which was detected and described before^21,22^; therefore, we propose that there will be both forms of exon 3 in combination with other ASVs, and that this combination will depend of the various alternative splicing regulatory mechanism such as RNA polymerase II elongation rate, SR and hnRNP protein balance, chromatin state etc.^24^. Our proposed HNF1B splicing pattern can be supported by the identification of the whole coding sequence of the HNF1BΔ8 transcript variant (containing a full length form of exon 3), which was analysed by sequencing and used during the optimization steps as described in the Methods section.

Intensive study of alternative splicing and its link to cancer in recent years has demonstrated that the natural splicing pattern of a variety of genes (*TP53, BCL2L1, FGFR2, EGFR, PTEN*, etc.) is influenced in many cancer types^25^. The formation of protein isoforms resulting from alternative splicing events has been documented many times as compromising the biological activity of the gene expression protein product and hence significantly influencing the natural gene function. In some cases, the specific ASV may impair the cellular homeostasis and thus potentially contribute to malignant transformation.

In this manuscript we described several HNF1B-predominant mRNA alternative splicing variants with an unknown functional impact and their diverse mRNA expression across different tissues. While protein isoforms of known mRNA variants 3p and Δ7_8 have already been described (3p: NM_001165923 - NP_001159395; Δ7_8: NM_001304286 - NP_001291215), the existence of protein isoforms derived from the novel predominant ASVs, which maintain the original open reading frame (Δ7 and Δ6_8), have yet to be confirmed. Moreover, we propose that the predominant ASVs with exon eight deletion (Δ5_8 and Δ8) could potentially create protein products (either with the combination of the canonical exon 3 or exon 3p variant, as mentioned above) in the same manner as the protein-coding ASV Δ7_8 (NP_001291215), in which the same type of frameshift in the last exon 9 was detected, leading to the formation of the alternative stop codon, located 92 bp after the canonical stop codon (Fig. 1).

Given the fact that variants Δ5_8; Δ6_8; Δ7; Δ7_8; Δ8 alter the coding sequence for the HNF1B C-terminus, which contains the domain with transactivation activity (located between exon 5 and 9) but preserves the coding sequences for dimerization and DNA-binding on the N-terminus (between exon 1 and 4; Fig. 2), we assume that the potential protein products of these splicing variants could have negative regulatory functions due to their preserved DNA binding and dimerization capacity, and a loss or reduction of transactivation activity with a potential impact on HNF1B-secured processes, if dysregulated.

Even though the data presented shows differences in tissue specific expression levels of the predominant variants in several of the analysed pools (3p in the EEC pool, Δ7_8 in the paired pancreas pools; etc.), the role of the predominant ASVs needs to be thoroughly investigated by suitable functional analysis on a protein level, prior to the final evaluation of their physiological functions. Nevertheless, based on cancer-specific splicing and our proposal of a HNF1B splicing pattern, we hypothesize that certain types of tumours can modify the splicing pattern of HNF1B and express ASVs with a significantly modified coding sequence on the C-terminus which results in proteins with an unknown, potentially regulatory function. Moreover, these protein isoforms cannot be distinguished using established immunohistochemical or other quantitative methods, due to the overall determination of HNF1B expression usually with the use of antibodies localized into the N-terminus (immunohistochemistry) or the primers localized into the 5′ part of mRNA (qPCR).

In conclusion, we revealed a series of HNF1B ASVs which have not yet been characterized. We believe that the identification and precise characterization of these variants is important and can represent a first step in the assessment of their biological meaning and significance for tumorigenesis in the several solid tumours in which HNF1B seems to be involved. Our data suggests the presence of several predominant ASVs with potentially different functions to canonical HNF1B and characterizes tissue- or disease-specific expression levels. However, the methodical approach used in this study has a semi-quantitative character, which does not allow us to evaluate the overall expression of HNF1B in the tissues analysed and calculate the expression ratios of the individual ASVs. The precise quantification of the canonical HNF1B transcript and identified HNF1B ASVs in different tissues must be performed on larger sample sets using methods such as ddPCR, qPCR or polyA-, capture free NGS methods with high coverage, prior to the evaluation of the potential functional impact of the respective ASVs.

## Material and Methods

### Patients and samples

**Figure 3:**
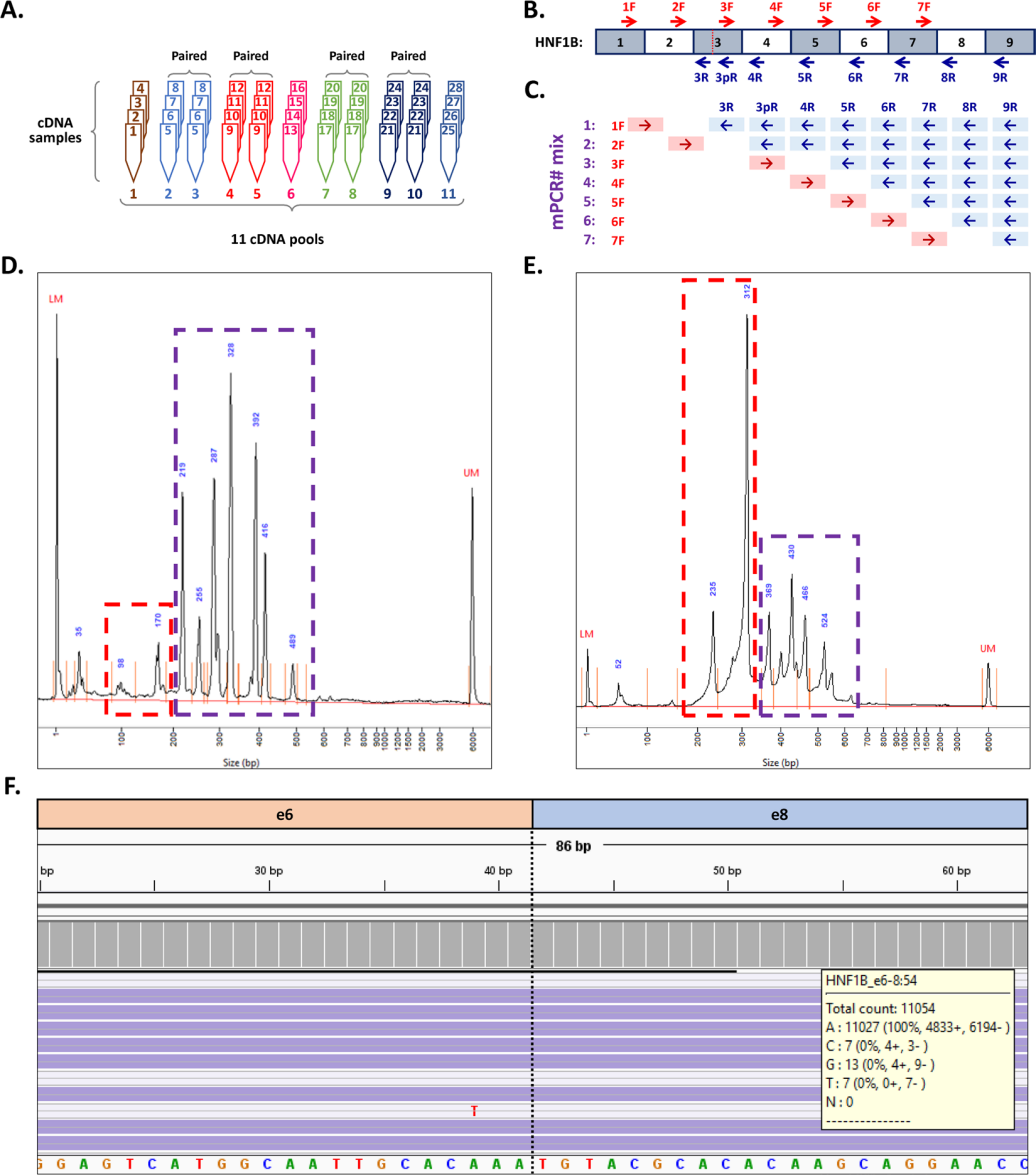
Overview of the methodical approach. (**A**) Scheme of the sample pools processing. cDNA samples from 32 individuals were mixed by four into 11 pools according to tissue type (1 – endometrial endometrioid carcinoma, 2 – colorectal carcinoma, 3 – paired colorectal healthy tissue, 4 – kidney carcinoma, 5 – paired healthy kidney, 6 – kidney oncocytoma, 7 – pancreatic carcinoma, 8 – paired healthy pancreas, 9 – prostate carcinoma, 10 – paired healthy tissue (seminal vesicles), 11 – prostate hyperplasia. (**B**) Scheme of the mPCR primer locations across the full-length HNF1B transcript (Red = forward primers; blue = reverse primers). In exon 3, an additional reverse primer was designed to cover the known HNF1B 3p variant (Fig. 1). (**C**) Scheme of 7 mPCR reactions with respective primer usage. The used mPCR reactions are designed to cover the amplification of all possible exon-exon junctions. (**D**) An example of the electropherograms from capillary electrophoresis of the final mPCR mixture of the healthy prostate pool. The red dashed line box represents the area of our interest with short amplicons raised from alternative splicing, the violet dashed line box represents the area with amplicons raised from the canonical transcripts. (**E**) Capillary electrophoresis of the prepared sequencing library of all mPCR pools. Same description applies for the dashed line boxes as for Fig. 2D. Comparing the size of the peaks between (**D** and **E**) shows enrichment of short amplicons after size selection (red dashed line box), and sequencing adaptor ligation adds +166 bp in amplicon length. (**F**) Manual analysis of the mapped reads in IGV viewer, example of the variant HNF1BΔ7. The left (red) part corresponds to the sequence of exon 6 and the right (blue) part of the amplicons corresponds to the sequence of exon 8; the number of reads was deducted and scored (the yellow pop-up box) using the grey IGV coverage bar.

A precise analysis of the HNF1B natural splicing pattern requires the careful selection of samples to avoid any possible bias in results. The greatest impact on the results can be caused by the presence of somatic or germline mutations localized in the splicing sites. In order to eliminate this effect, only the samples with no mutations in *HNF1B* consensus splicing sites and adjacent areas (±15 bp) were selected (*HNF1B* mutation analysis was performed during a parallel project; data not shown). Moreover, we minimized the effect of potential private mutations in deep intronic splicing regulation sites (which are difficult to analyse and interpret) by compiling the tissue sample pools. Each tissue sample pool was created by the equimolar mixing of four cDNA samples of the same tissue type. Altogether, 11 tissue pools were analysed: 1 - endometrial endometrioid carcinoma (EEC); 2 - colorectal carcinoma and 3 - paired healthy tissue (full thickness cross-section of the wall of non-tumour large intestine); 4 - kidney carcinoma and 5 - paired healthy tissue (both non-tumour kidney medulla and cortex tissue); 6 - kidney oncocytoma; 7 - pancreatic carcinoma and 8 - paired healthy tissue (distant parts of the resected pancreas); 9 - prostate carcinoma and 10 - paired healthy tissue (seminal vesicles) and 11 - prostate hyperplasia (Fig. 3A). Tissue samples were collected by trained pathologists who macroscopically evaluated the whole resected tissue specimens in their native state (after surgical procedure, prior to fixation) and then took a sample of representative tumour and non-tumour tissue for storage. The non-tumour tissue, which was considered as healthy tissue, was taken from the periphery of each resected organ specimen.

Each of the paired tumour and healthy tissue pools were compiled from samples obtained from the same four individuals, as such 11 specific tissue sample pools were created from the total of 44 tissue samples, gained from 28 individuals (Fig. 3A).

The samples were provided by The Bank of Biological Material, First Faculty of Medicine, Charles University, and stored in RNAlater according to the manufacturer’s instructions (Thermo Fisher) until the genetic material was isolated.

### Ethics statement

The study has been approved by The Ethics Committee of General University Hospital in Prague in compliance with the Helsinki Declaration (ethical approval number 41/16 as a part of the grant from the Czech Research Council 17-28404A) and all experiments were performed in accordance with these guidelines and regulations. The ethics committee which approved this study waived the need for informed consent.

### Total RNA and DNA isolation, quality control and cDNA synthesis

All RNA samples were processed according to MIQE guidelines^26^. The thawed samples (10–30 mg) were homogenized using MagNA Lyser Green Beads tubes in a MagNA Lyser Instrument (Roche) in the presence of 600 µl of RLT Plus buffer (Qiagen) with 6 µl of 14.3 M 2-mercaptoethanol (Sigma-Aldrich). The total RNAs and DNAs were isolated according to the Simultaneous Purification of Genomic DNA and Total RNA from Animal Tissues protocol by using an AllPrep DNA/RNA Mini kit (Qiagen). All samples were quantified by NanoDrop 2000 (Thermo Fisher), and the RNA samples were additionally characterized by an RNA Quality Number (RQN) using Fragment Analyzer (AATI) capillary electrophoresis system and Standard RNA kit (AATI; tissue samples RQN_mean_ = 9.7; range 7.5–10).

All RNA samples were treated by DNase I (Thermo Fisher) and cDNA was synthetized from 1 µg of total RNA in 20 µl reaction using SuperScript III Reverse Transcriptase (Thermo Fisher) with random hexamers (Roche) as described previously^27^. A routine qPCR control of cDNA quality/integrity was performed using *GAPDH* primers (from RevertAid H Minus First Strand cDNA Synthesis Kit; Thermo Fisher) and FirePol EvaGreen HRM Mix (Solis Biodyne) according to the manufacturer’s instructions on LightCycler II (Roche). The resulting crossing points (Cp) of all the amplified 496 bp *GAPDH* amplicons ranged between 17.8 and 21.7; the specificity of each amplicon was verified by HRM (high resolution melting) analysis and capillary electrophoresis (Supplementary Fig. 1). Moreover, all samples included in the study were briefly analysed for *HNF1B* gene expression by the same qPCR protocol as mentioned above (with use of FirePol EvaGreen HRM Mix protocol and LightCycler II instrument) except for the primers localized in the 5′ UTR of the *HNF1B* gene (Supplementary Table 1). The resulting crossing points showed the expression of HNF1B transcripts in all samples (Cp min – 22.0; max – 29.3; median – 24.3; Supplementary Fig. 2A), the specificity of each amplicon was verified by HRM analysis (Supplementary Fig. 2B) and Sanger sequencing (data not shown).

### Multiplex PCR primer design

Primers for multiplex PCR (mPCR) were designed in order to cover every possible exon-exon junction of all canonical and known alternative transcript isoforms, as previously described^27^. Such primer design, in combination with the short PCR elongation step, leads to the preferential formation of small mPCR amplicons (~80–100 bp) from alternatively spliced exon-exon junctions, while the amplification of the canonical exon-exon junctions (with a longer mPCR product) is suppressed.

For HNF1B transcripts (NM_000458, NM_001165923), 15 primers (Fig. 3B) – 7 forward and 8 reverse – were used for the amplification of all of the exon-exon junctions in 7 mPCR reactions per one cDNA pool (Fig. 3C, Supplementary Table 1). All individual PCRs were optimized separately (Supplementary Fig. 3) using plasmid containing a HNF1BΔ8 coding sequence (HNF1B exon 8 deletion) and a native cDNA test sample as templates (cDNAs for testing were prepared from healthy ovarian tissues as described in RNA isolation and cDNA synthesis). The insert sequence for the control plasmid was constructed by PCR amplification of the cDNA template with primers HNF1B e1F and HNF1B e9R (Supplementary Table 1), using 5x HOT FIREPol EvaGreen HRM Mix (Solis BioDyne) according to the manufacturer’s protocol (12-minute incubation at 95 °C followed by 35 cycles of 95 °C for 15 s, 60 °C for 20 s and 72 °C for 2 min and a final 5 min extension at 72 °C), and subjected to ligation into the pCR 2.1 TA cloning vector (Invitrogen) according to the manufacturer’s instructions. The prepared vector was transformed into the TOP10F- chemically competent cells (Invitrogen) and plated onto LB plates containing ampicillin, X-Gal, and IPTG. Blue/white screening-positive single cell clones were fully sequenced, amplified in the above-mentioned competent cells, and MIDI prepared using Qiagen plasmid MIDI isolation kit (Qiagen). The coding sequence of the full-length HNF1BΔ8 transcript was detected in the plasmid insert and confirmed by the standard capillary Sanger sequencing (data not shown).

Each mPCR reaction (40 µl) contained one forward primer (4.5 pmol) and a set of respective reverse primers (1.5 each; Fig. 3C) with 4 µl of template cDNA (equivalent to 200 ng RNA). The FastStart High Fidelity PCR System (Roche) was used for the amplification according to the manufacturer’s instructions, which involved 4-minute incubation at 95 °C followed by 35 cycles (95 °C for 10 s, 60 °C for 20 s and 72 °C for 10 s), and a final extension (72 °C for 5 min). After amplification, 6 µl of each of 7 mPCR reactions, per respective cDNA pool, were mixed back together. The profiles of the resulting mPCR pools, which correspond to the original 11 cDNA tissue pools, were characterized by capillary electrophoresis using a High Sensitivity NGS Fragment Analysis Kit (AATI) and Fragment Analyzer Instrument (AATI; Fig. 3D, Supplementary Fig. 4A).

### Size Selection

In order to reduce large amplicons containing canonical exon-exon junctions (>150 bp), and to purify the remaining shorter amplicons, all mPCR pools were subjected to a two-step size selection using AMPure XP reagent (Beckman Coulter). In the first step a 1.8x reagent concentration, and in the second step a 3x reagent concentration was used. The size selection process resulted in the purification and enrichment of short 80–150 bp amplicons in the mPCR pools, while the number of large amplicons was reduced (Fig. 3D,E).

### NGS library preparation and sequencing

Purified mPCR pools were processed for NGS sequencing using KAPA Hyper Prep Kit (Kapa, Roche) according to the manufacturer’s instructions. The library preparation included the following steps: amplicons end repair, 3′dATP-tailing, ligation with Illumina sequencing adaptors, sample purifications (0.8x concentration of AMPure reagent), and 7 cycle PCR amplification with primers containing index sequences (TruSeq HT – double index approach), unique for each mPCR pool. After PCR amplification, libraries of mPCR pools were purified (1x AMPure XP concentration), quantified by Qubit HS DNA kit (Thermo Fisher), and characterized by capillary electrophoresis (High Sensitivity NGS Fragment Analysis Kit; Fragment Analyzer; AATI; Supplementary Fig. 4B).

In order to achieve a high sample diversity and deep sequencing coverage, the mPCR-prepared libraries were pooled with routine high-complex panel libraries in a standard NextSeq run (Mid-output kit v2, 150 cycles; Illumina). Each mPCR library covered ~1% of the sequencing run capacity.

Additionally, the created mPCR libraries were sequenced by MiSeq kit v2 (300 cycles; Illumina) to be able to identify possible longer (>150 bp) insertions due to longer reads. Sequencing was performed in the presence of PhiX (~30%). Each mPCR library covered ~6% of the sequencing capacity, which resulted in a sufficient sensitivity for the detection of long amplicons.

### Bioinformatics

Bioinformatical analyses were performed using the NextGENe software v2.4.2 (Softgenetics) in the same way for both NextSeq and MiSeq runs. Raw sequencing data were demultiplexed, universal adaptors were trimmed, and reads with a low quality (median score <25; number of uncalled bases ≥3; number of called bases <40) were removed. For the ASVs identification, the remaining reads were mapped in a double-step approach as previously described^27^. For the first mapping the synthetic FASTA file (Supplementary Table 2), with all the possible and known alternative exon-exon junctions, was used as a template (alignment settings – matching base percentage = 95%, including indel detection). In this step, all cassette (exon) or multicasette (multiexon) deletions, and a majority of splice donor/acceptor site shifts (SDS/SAS) were identified. In the second mapping step the reads were mapped onto the genomic sequence of *HNF1B* (NG_013019.2; alignment settings – matching base percentage = 40%, including indel detection) for the detection of intron exonization (retention) and large SDS/SAS identification. All the mapping results were manually analysed in IGV (Broad Institute; Fig. 3F)^28^. Only those counts of the true mapped reads in each splicing event were reported (splicing events without 100% coverage of at least one paired read were excluded).

After comparing the data gained on the basis of the short reads from NextSeq (2 × 75 bp), and the longer reads from MiSeq (2 × 150 bp) we decided to proceed with calculating the exon deletions and SDS/SAS shifts based on the NextSeq data, which showed better sensitivity when using the synthetic FASTA file. The intron exonizations were calculated based on the MiSeq longer reads, where multiple exonizations were detected and fully characterized (in contrast with the NextSeq shorter reads).

To compare the raw numbers of the ASV reads in the examined mPCR pools (Supplementary Table 3), the total number of sequencing reads of each mPCR library pool (including canonical exon-exon junctions corresponding to reference sequences, detected ASVs and minor non-specific sequences) were normalized to 10^6^ of all sequenced reads and individual ASVs read counts were then recalculated accordingly. The recalculated read numbers characterize the portion of a single ASV (read number of individual variants per one million total reads) in the respective tissue pool.

Normalized read counts were used for the comparison of single variant relative expression within the analysed tissue pools. Our previous study^27^, describing and validating the methodic approach used, confirmed the reliable reproducibility of ASVs detection for variants with a normalized reads count >100 (0.01% of normalized reads). Due to the unfailing characterization of HNF1B ASVs, we discussed and considered only variants with a read count >1000 (0.1% of normalized reads) for further semi-quantitative evaluation of the detected variants within the analysed tissue pools.

### Nomenclature used for the description of the splicing variants

The splicing variants were divided into four main categories: (a) deletion/exon skipping/intronization (marked as “Δ”) of one whole canonical exon (sometimes denoted as “cassette”; “CΔ”), or more canonical exons in a row (sometimes denoted as “multicasette”; “mCΔ”); (b) insertion/intron retention/exonization (marked as “▼”) of the internal canonical intronic region (sometimes denoted as “cassette”; “C▼”); (c) splice acceptor shift (on the 5′ part of the exon; marked as “p”; sometimes as SAS) which can result in both the deletion/intronization of the 5′ part of the exon sequence (SASΔ), or insertion/exonization of the adjacent intronic sequence to the 5′ part of the exon (SAS▼); (d) splice donor shift (on the 3′ exon splicing site; marked as “q”; sometimes SDS), which can result in both the deletion/intronization of the 3′ part of the exon sequence (SDSΔ) or the insertion/exonization of the adjacent intronic sequence to the 3′part of the exon (SDS▼).

## Supplementary information

Supplementary Information.

## Data Availability

The source data generated during and/or analysed during the current study are included in this published article (and its Supplementary Information Files) or are available from the corresponding author on reasonable request.
