# Supplementary material for: Identification of novel HNF1B mRNA splicing variants and their qualitative and semi-quantitative profile in selected healthy and tumour tissues
Source: Sci Rep. 2020 Apr 24;10:6958. doi: 10.1038/s41598-020-63733-x (PMC7181708; doi:10.1038/s41598-020-63733-x)

**Supplementary Information**

**Identification of novel HNF1B mRNA splicing variants and their qualitative and semi-quantitative profile in selected healthy and tumour tissues**

Running title: HNF1B alternative splicing variants

Jan Hojny^1,2†^, Michaela Bartu^1,2†^, Eva Krkavcova^1^, Kristyna Nemejcova^1^, Jan Sevcik^3^, David Cibula^4^, Vladimir Fryba^5^, Lenka Plincelnerova^6^, Pavel Dundr^1^, Ivana Struzinska^1^*

^1^Institute of Pathology, First Faculty of Medicine, Charles University and General University Hospital in Prague, 12808, Czech Republic

^2^First Faculty of Medicine, Charles University, Prague, 12108, Czech Republic

^3^Institute of Biochemistry and Experimental Oncology, First Faculty of Medicine, Charles University, Prague, 12108, Czech Republic

^4^Gynecological Oncology Center, Department of Obstetrics and Gynecology, First Faculty of Medicine, Charles University and General University Hospital in Prague, 12808, Czech Republic

^5^1st Department of Surgery - Department of Abdominal, Thoracic Surgery and Traumatology, First Faculty of Medicine, Charles University and General University Hospital, Prague, 12808, Czech Republic

^6^Department of Urology, First Faculty of Medicine, Charles University and General University Hospital in Prague, 12808, Czech Republic

†These authors contributed equally to this work

*email: [ivana.struzinska@vfn.cz](mailto:ivana.struzinska@vfn.cz)

Correspondence:

Tel: +420224968685
Ivana Struzinska, Ph.D., Institute of Pathology, First Faculty of Medicine, Charles University and General University Hospital in Prague, Studnickova 2, 12800, Czech Republic

**Supplementary figure 1.** Control amplification of 496 bp GAPDH amplicon in all 44 cDNA samples in the study. A) Amplification curves of GAPDH amplicons (Cp ranged between 17.8 and 21.7; Roche Light Cycler 480 II Software). B) Respective melting peaks of all cDNA GAPDH amplicons (Roche Light Cycler 480 II Software). C) Capillary electrophoresis of randomly selected 496 bp GAPDH amplicon. LM = lower marker (1 bp); UM = upper marker (6000 bp); High Sensitivity NGS Fragment Analysis Kit (AATI); Fragment Analyzer (AATI).


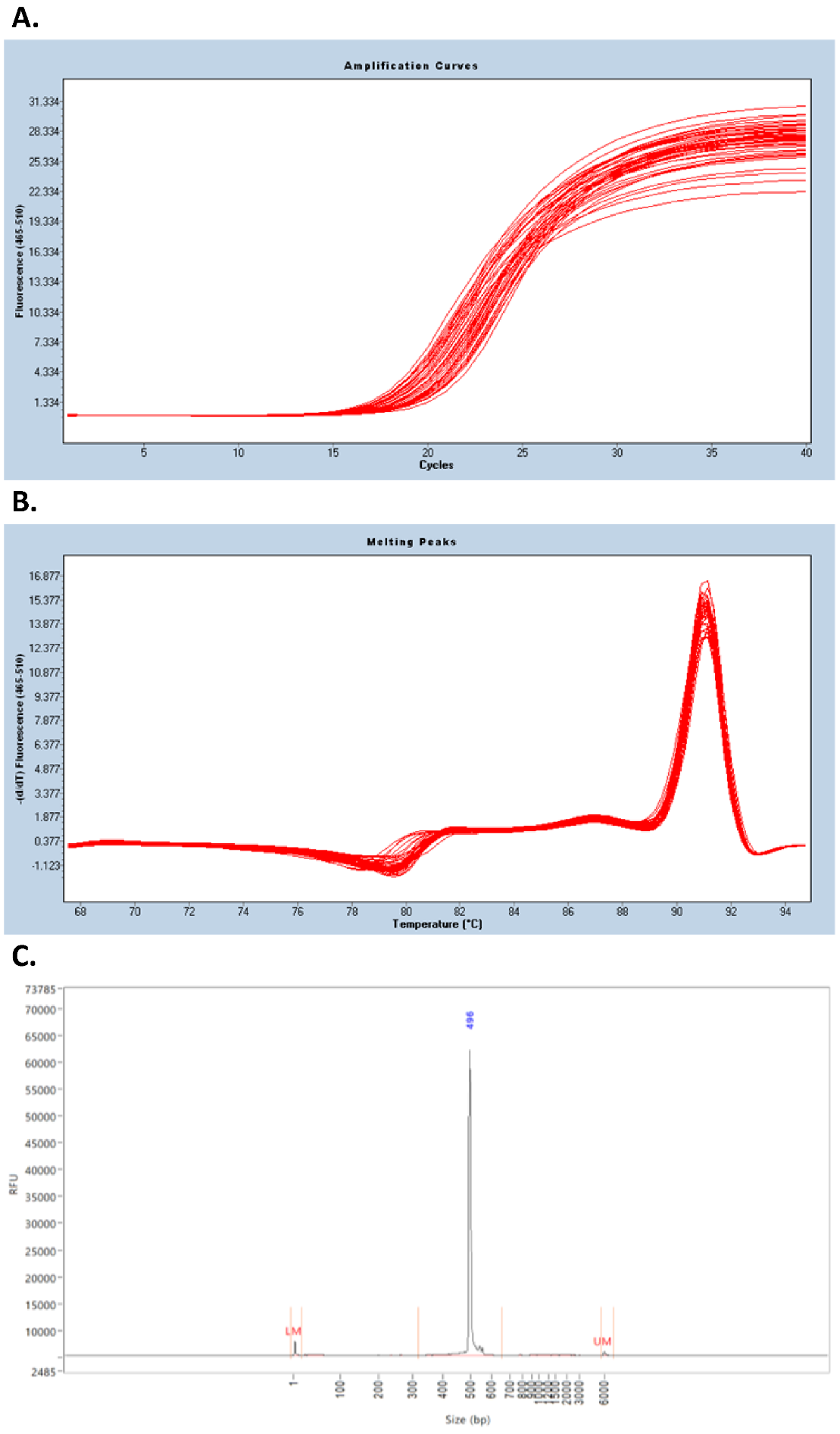


**Supplementary figure 2.** Control amplification of 111 bp HNF1B 5’ UTR amplicon in all 44 cDNA samples in the study. A) Amplification curves of GAPDH amplicons (Cp ranged between 22.0 and 29.3; Roche Light Cycler 480 II Software). B) Respective melting peaks of all cDNA 5’ UTR amplicons (Roche Light Cycler 480 II Software).


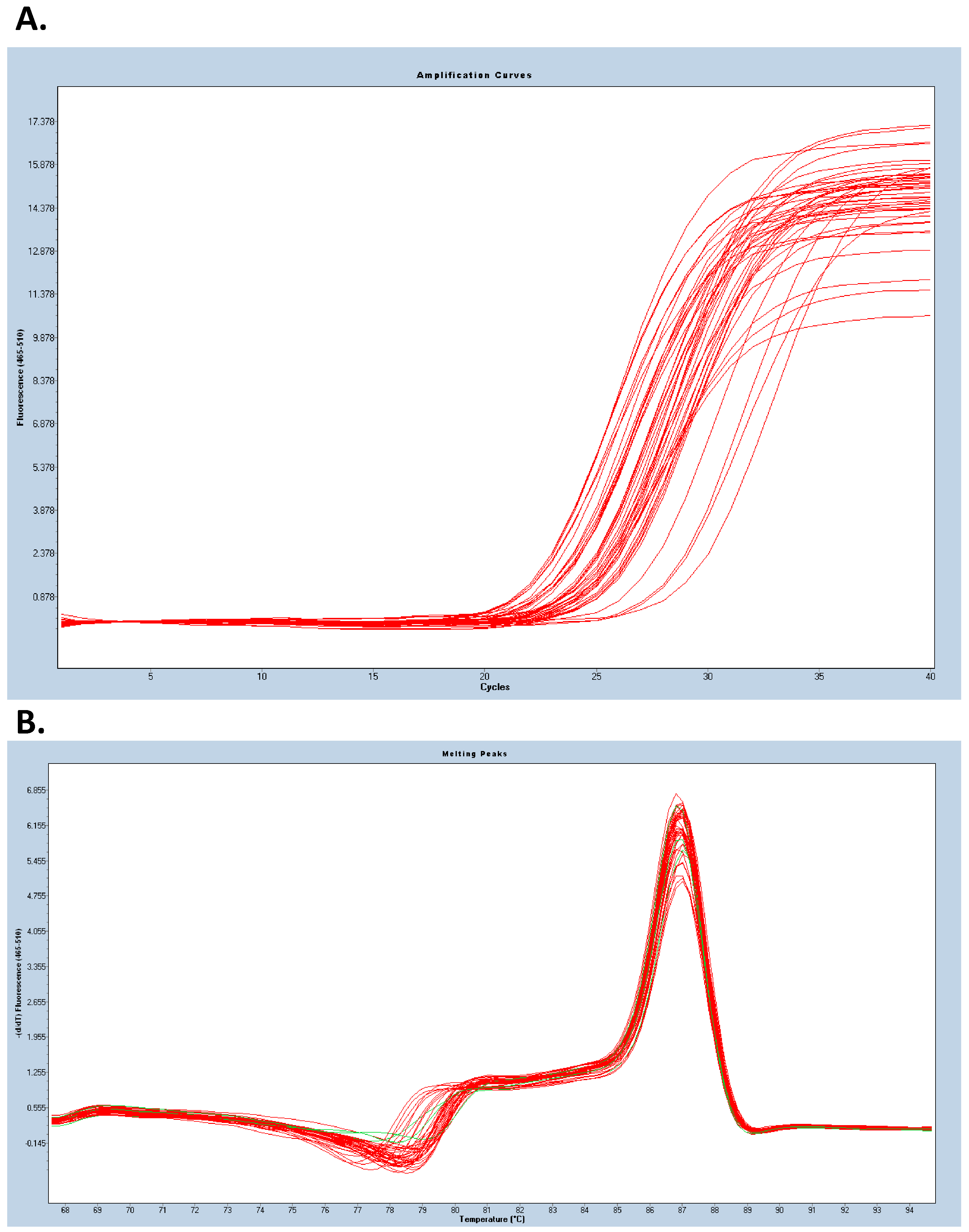


**Supplementary figure 3.** Primer optimization in individual PCR reactions visualized by UV-light after electrophoretic separation in 1% agarose gel. For majority of primers, HNF1BΔ8 plasmid insert was used as a template. Amplicons with HNF1B 8R primer were optimized separately using cDNA test sample (healthy ovary tissue) due to the detected deletion of exon 8 in plasmid insert sequence. A) PCR products raised from plasmid HNF1BΔ8 template and appropriate HNF1B primer pairs (red – forward primer; blue – set of respective reverse primers). Red stars indicate non-specific amplicons. B) PCR products raised from cDNA test sample and set of HNF1B 8R respective primers, unsuccessful amplification using HNF1B 1F and HNF1B 8R primers was repeated. Individual photos of gels (A, B, and C) were cropped from the surroundings, transformed to negative and then to grey colour scale with minor brightness or contrast adjustment by using ACD Photo Editor 3.1 (ACD Systems, Ltd.). Original photos of gels are included at the end of this Supplementary information file.


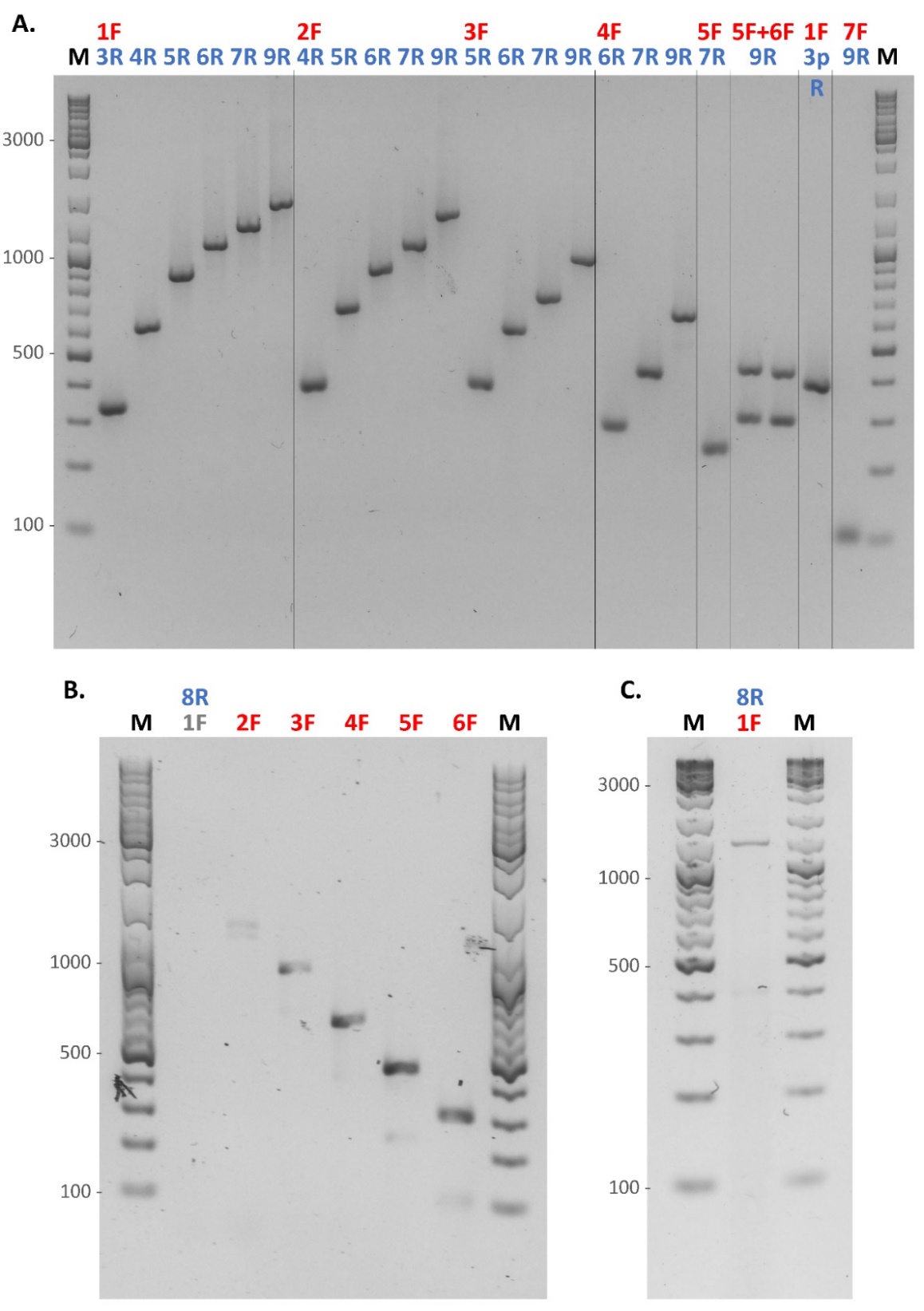


**Supplementary figure 4.** Comparison of electropherograms of PCR products after multiplex PCR (mPCR) and after size selection and NGS library preparation. A) All 11 cDNA pools after mPCR reactions. B) All 11 cDNA pools after library preparation. Peaks at position 1 and 6000 are alignment markers. Capillary electrophoresis was performed on Fragment Analyzer (AATI) using High Sensitivity NGS Fragment Analysis Kit (AATI).


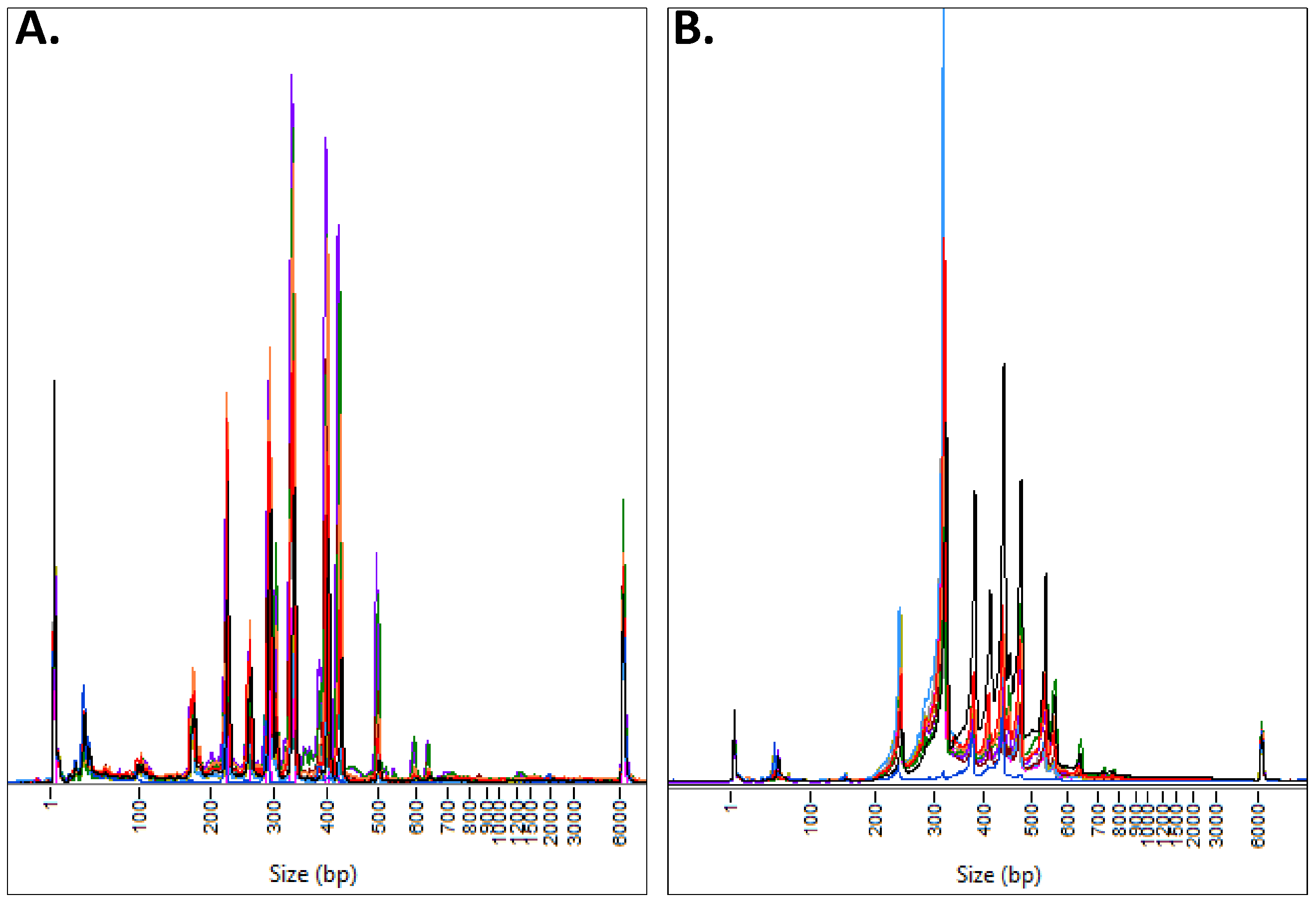


**Supplementary table 1**

| Primer name | Sequence (5' → 3') | Primer lenght (bp) | **T_annealing_ (°C)** | % GC | Distance to exon end without primer (bp) | Distance to exon end including primer (bp) |
| --- | --- | --- | --- | --- | --- | --- |
| HNF1B 1F | GCGCTCAACACCGAGGAG | 18 | **57,6** | 66,7 | 38 | 56 |
| HNF1B 2F | GCTCTGTACACCTGGTACGTC | 21 | **56,5** | 57,1 | 25 | 46 |
| HNF1B 3F | CCCAGCAAGGAAGAGAGAGAGG | 22 | **57,9** | 59,1 | 22 | 44 |
| HNF1B 4F | CACCACCAGCCCAGCTC | 17 | **56,9** | 70,6 | 23 | 40 |
| HNF1B 5F | GACCCAGGCCACAATCTCC | 19 | **56,6** | 63,2 | 20 | 39 |
| HNF1B 6F | CATCATGACACCCCTCTCTGG | 21 | **56,1** | 57,1 | 20 | 41 |
| HNF1B 7F | CCAGCAGCCCTTCATGGC | 18 | **57,8** | 66,7 | 29 | 47 |
|  |  |  |  |  |  |  |
| HNF1B 3R | CAGCAGCTGATCCTGACTGC | 20 | **57,5** | 60 | 45 | 65 |
| HNF1B 3pR | CATCATCGGACTGCCCAGG | 19 | **56,8** | 63,2 | 23 | 42 |
| HNF1B 4R | AGGCCGTGGGCTTTGG | 16 | **56,6** | 68,8 | 32 | 48 |
| HNF1B 5R | GGAGGAAGTGATCTCATTGTTTCCC | 25 | **57,2** | 48 | 19 | 44 |
| HNF1B 6R | GATATTCGTCAAGGTGCTGACTGG | 24 | **57,5** | 50 | 27 | 51 |
| HNF1B 7R | GGCCACACTGTTGATGACAGG | 21 | **57,8** | 57,1 | 29 | 50 |
| HNF1B 8R | GGGAGGTGTGGGAATACTGGG | 21 | **58** | 61,9 | 24 | 45 |
| HNF1B 9R | GCACGAAGTAAGTGGTGTGTG | 21 | **56,1** | 52,4 | 25 | 46 |
|  |  |  |  |  |  |  |
| HNF1B 5UTR F | CATGGCAAGTTAGAAGTTTTCTGACTCC | 28 | **58,1** | 42,9 | - | - |
| HNF1B 5UTR R | GCAAACCCCAAATCCAGGAACC | 22 | **58,4** | 54,5 | - | - |

**Supplementary table 1.** List of primer sequences including primer length, annealing temperature (Tan; calculated by AnnHyb v.4.946; http://bioinformatics.org/annhyb), percentage of G and C nucleotides in designed primer sequence (% GC) and distance to 3’ exon ends (forward primer) or 5’ exon ends (reverse primers) with or without primer sequences.

**Supplementary table 2**

| Origin of fasta sequence | Name of fasta sequence | Sequence |
| --- | --- | --- |
| Hypothesized | >HNF1B_e1-2 | CGGCGACGACTATGACACACCTCCCATCCTCAAGGAGCTGCAGGCGCTCAACACCGAGGAGGCGGCGGAGCAGCGGGCGGAGGTGGACCGGATGCTCAGTGAGGACCCTTGGAGGGCTGCTAAAATGATCAAGGGTTACATGCAGCAACACA |
| Hypothesized | >HNF1B_e2-3p | GCTCTGTACACCTGGTACGTCAGAAAGCAACGAGAGATCCTCCGACAGTTCAGTCAACAGAGCCATGGGCCTGGGCAGTCCGATGATG |
| Hypothesized | >HNF1B_e2-3 | GCTCTGTACACCTGGTACGTCAGAAAGCAACGAGAGATCCTCCGACAATTCAACCAGACAGTCCAGAGTTCTGGAAATATGACAGACAAAAGCAGTCAGGATCAGCTGCTG |
| Hypothesized | >HNF1B_e3-4 | CCCAGCAAGGAAGAGAGAGAGGCCTTAGTGGAGGAATGCAACAGGGCAGAATGTTTGCAGCGAGGGGTGTCCCCCTCCAAAGCCCACGGCCT |
| Hypothesized | >HNF1B_e4-5 | CACCACCAGCCCAGCTCCTCTCCTCCAAACAAGCTGTCAGGAGTGCGCTACAGCCAGCAGGGAAACAATGAGATCACTTCCTCC |
| Hypothesized | >HNF1B_e5-6 | GACCCAGGCCACAATCTCCTCTCACCTGATGGTAAAATGATCTCAGTCTCAGGAGGAGGTTTGCCCCCAGTCAGCACCTTGACGAATATC |
| Hypothesized | >HNF1B_e6-7 | CATCATGACACCCCTCTCTGGAGTCATGGCAATTGCACAAAGCCTCAACACCTCCCAAGCACAGAGTGTCCCTGTCATCAACAGTGTGGCC |
| Hypothesized | >HNF1B_e7-8 | CCAGCAGCCCTTCATGGCAGCTGTGACTCAGCTGCAGAACTCACACATGTACGCACACAAGCAGGAACCCCCCCAGTATTCCCACACCTCCC |
| Hypothesized | >HNF1B_e8-9 | GCAGGAACCCCCCCAGTATTCCCACACCTCCCGGTTTCCATCTGCAATGGTGGTCACAGATACCAGCAGCATCAGTACACTCACCAACATGTCTTCAAGTAAACAGTGTCCTCTACAAGCCTGGTGATGCCCACACACCACTTACTTCGTGC |
| Hypothesized | >HNF1B_del2,3p | GCGCTCAACACCGAGGAGGCGGCGGAGCAGCGGGCGGAGGTGGACCGGATGCTCAGAGTTCAGTCAACAGAGCCATGGGCCTGGGCAGTCCGATGATG |
| Hypothesized | >HNF1B_del2 | GCGCTCAACACCGAGGAGGCGGCGGAGCAGCGGGCGGAGGTGGACCGGATGCTCAGAATTCAACCAGACAGTCCAGAGTTCTGGAAATATGACAGACAAAAGCAGTCAGGATCAGCTGCTG |
| Hypothesized | >HNF1B_del2_3 | GCGCTCAACACCGAGGAGGCGGCGGAGCAGCGGGCGGAGGTGGACCGGATGCTCAGGGCAGAATGTTTGCAGCGAGGGGTGTCCCCCTCCAAAGCCCACGGCCT |
| Hypothesized | >HNF1B_del2_4 | GCGCTCAACACCGAGGAGGCGGCGGAGCAGCGGGCGGAGGTGGACCGGATGCTCAGGAGTGCGCTACAGCCAGCAGGGAAACAATGAGATCACTTCCTCC |
| Hypothesized | >HNF1B_del2_5 | GCGCTCAACACCGAGGAGGCGGCGGAGCAGCGGGCGGAGGTGGACCGGATGCTCAGATCTCAGTCTCAGGAGGAGGTTTGCCCCCAGTCAGCACCTTGACGAATATC |
| Hypothesized | >HNF1B_del2_6 | GCGCTCAACACCGAGGAGGCGGCGGAGCAGCGGGCGGAGGTGGACCGGATGCTCAGGCCTCAACACCTCCCAAGCACAGAGTGTCCCTGTCATCAACAGTGTGGCC |
| Hypothesized | >HNF1B_del2_7 | GCGCTCAACACCGAGGAGGCGGCGGAGCAGCGGGCGGAGGTGGACCGGATGCTCAGTGTACGCACACAAGCAGGAACCCCCCCAGTATTCCCACACCTCCC |
| Hypothesized | >HNF1B_del2_8 | GCGCTCAACACCGAGGAGGCGGCGGAGCAGCGGGCGGAGGTGGACCGGATGCTCAGTGTCCTCTACAAGCCTGGTGATGCCCACACACCACTTACTTCGTGC |
| Hypothesized | >HNF1B_del3 | GCTCTGTACACCTGGTACGTCAGAAAGCAACGAGAGATCCTCCGACGGCAGAATGTTTGCAGCGAGGGGTGTCCCCCTCCAAAGCCCACGGCCT |
| Hypothesized | >HNF1B_del3_4 | GCTCTGTACACCTGGTACGTCAGAAAGCAACGAGAGATCCTCCGACGAGTGCGCTACAGCCAGCAGGGAAACAATGAGATCACTTCCTCC |
| Hypothesized | >HNF1B_del3_5 | GCTCTGTACACCTGGTACGTCAGAAAGCAACGAGAGATCCTCCGACATCTCAGTCTCAGGAGGAGGTTTGCCCCCAGTCAGCACCTTGACGAATATC |
| Hypothesized | >HNF1B_del3_6 | GCTCTGTACACCTGGTACGTCAGAAAGCAACGAGAGATCCTCCGACGCCTCAACACCTCCCAAGCACAGAGTGTCCCTGTCATCAACAGTGTGGCC |
| Hypothesized | >HNF1B_del3_7 | GCTCTGTACACCTGGTACGTCAGAAAGCAACGAGAGATCCTCCGACTGTACGCACACAAGCAGGAACCCCCCCAGTATTCCCACACCTCCC |
| Hypothesized | >HNF1B_del3_8 | GCTCTGTACACCTGGTACGTCAGAAAGCAACGAGAGATCCTCCGACTGTCCTCTACAAGCCTGGTGATGCCCACACACCACTTACTTCGTGC |
| Hypothesized | >HNF1B_del4 | CCCAGCAAGGAAGAGAGAGAGGCCTTAGTGGAGGAATGCAACAGGAGTGCGCTACAGCCAGCAGGGAAACAATGAGATCACTTCCTCC |
| Hypothesized | >HNF1B_del4_5 | CCCAGCAAGGAAGAGAGAGAGGCCTTAGTGGAGGAATGCAACAGATCTCAGTCTCAGGAGGAGGTTTGCCCCCAGTCAGCACCTTGACGAATATC |
| Hypothesized | >HNF1B_del4_6 | CCCAGCAAGGAAGAGAGAGAGGCCTTAGTGGAGGAATGCAACAGGCCTCAACACCTCCCAAGCACAGAGTGTCCCTGTCATCAACAGTGTGGCC |
| Hypothesized | >HNF1B_del4_7 | CCCAGCAAGGAAGAGAGAGAGGCCTTAGTGGAGGAATGCAACAGTGTACGCACACAAGCAGGAACCCCCCCAGTATTCCCACACCTCCC |
| Hypothesized | >HNF1B_del4_8 | CCCAGCAAGGAAGAGAGAGAGGCCTTAGTGGAGGAATGCAACAGTGTCCTCTACAAGCCTGGTGATGCCCACACACCACTTACTTCGTGC |
| Hypothesized | >HNF1B_del5 | CACCACCAGCCCAGCTCCTCTCCTCCAAACAAGCTGTCAGATCTCAGTCTCAGGAGGAGGTTTGCCCCCAGTCAGCACCTTGACGAATATC |
| Hypothesized | >HNF1B_del5_6 | CACCACCAGCCCAGCTCCTCTCCTCCAAACAAGCTGTCAGGCCTCAACACCTCCCAAGCACAGAGTGTCCCTGTCATCAACAGTGTGGCC |
| Hypothesized | >HNF1B_del5_7 | CACCACCAGCCCAGCTCCTCTCCTCCAAACAAGCTGTCAGTGTACGCACACAAGCAGGAACCCCCCCAGTATTCCCACACCTCCC |
| Hypothesized | >HNF1B_del5_8 | CACCACCAGCCCAGCTCCTCTCCTCCAAACAAGCTGTCAGTGTCCTCTACAAGCCTGGTGATGCCCACACACCACTTACTTCGTGC |
| Hypothesized | >HNF1B_del6 | GACCCAGGCCACAATCTCCTCTCACCTGATGGTAAAATGGCCTCAACACCTCCCAAGCACAGAGTGTCCCTGTCATCAACAGTGTGGCC |
| Hypothesized | >HNF1B_del6_7 | GACCCAGGCCACAATCTCCTCTCACCTGATGGTAAAATGTGTACGCACACAAGCAGGAACCCCCCCAGTATTCCCACACCTCCC |
| Hypothesized | >HNF1B_del6_8 | GACCCAGGCCACAATCTCCTCTCACCTGATGGTAAAATGTGTCCTCTACAAGCCTGGTGATGCCCACACACCACTTACTTCGTGC |
| Hypothesized | >HNF1B_del7 | CATCATGACACCCCTCTCTGGAGTCATGGCAATTGCACAAATGTACGCACACAAGCAGGAACCCCCCCAGTATTCCCACACCTCCC |
| Hypothesized | >HNF1B_del7_8 | CATCATGACACCCCTCTCTGGAGTCATGGCAATTGCACAAATGTCCTCTACAAGCCTGGTGATGCCCACACACCACTTACTTCGTGC |
| Hypothesized | >HNF1B_del8 | CCAGCAGCCCTTCATGGCAGCTGTGACTCAGCTGCAGAACTCACACATGTCCTCTACAAGCCTGGTGATGCCCACACACCACTTACTTCGTGC |
| Identified by whole gene mapping | >HNF1B_i2_ins106 | GCTCTGTACACCTGGTACGTCAGAAAGCAACGAGAGATCCTCCGACagtgcttagcctccatgtttattcatctttaaaatggaattaatcactcctgatgagccctgcttcatgcagatgtgaagacgaggacagcaaagctttcttcttcAATTCAACCAGACAGTCCAGAGTTCTGGAAATATGACAGACAAAAGCAGTCAGGATCAGCTGCTGTTTCTCTTTCCAGAGTTCAGTCAACAGAGCCATGGGCCTGGGCAGTCCGATGATG |
| Identified by whole gene mapping | >HNF1B_i4_ins157bp | CACCACCAGCCCAGCTCCTCTCCTCCAAACAAGCTGTCAGgtgttttatggatggagacagaaatgagcggccgttcttcttcattgtctacaaccctacagccaggggaactagagggtttgcatccctacctgtaaacttcaatctcaccgtgaagaagaaggtccctgaagtctctgaacaccttgctctgtatGAGTGCGCTACAGCCAGCAGGGAAACAATGAGATCACTTCCTCC |
| Identified by whole gene mapping | >HNF1B_i4_ins94bp | CACCACCAGCCCAGCTCCTCTCCTCCAAACAAGCTGTCAGccctcttaagctctaggtcatatcggccttgggtaaagtccttaggaagatccaaggaaaactggcttggggctcatctagtctcatcttgGAGTGCGCTACAGCCAGCAGGGAAACAATGAGATCACTTCCTCC |
| Identified by whole gene mapping | >HNF1B_i4_ins128bp | CACCACCAGCCCAGCTCCTCTCCTCCAAACAAGCTGTCAGagcaaactccagtggtttactaaacatccacatctgcctgtgtgtggtgctgcctgccccacctcccagcagacacacctttccctgtgccctggaaggcagccaaatgggacccccagcaaaggaatGAGTGCGCTACAGCCAGCAGGGAAACAATGAGATCACTTCCTCC |
| Identified by whole gene mapping | >HNF1B_i5_ins148 | GACCCAGGCCACAATCTCCTCTCACCTGATGGTAAAATGgggagggaggagccgcagattcccgactttcatgctgaaggtggagctgcacacattcccagagtgggggccaggatgggtgagggcaacagggtgcagaggccattttagatcttgacgtgtgtgctgtgtaggcaacacagaaacgATCTCAGTCTCAGGAGGAGGTTTGCCCCCAGTCAGCACCTTGACGAATATC |
| Identified by whole gene mapping | >HNF1B_i6_ins92 | CATCATGACACCCCTCTCTGGAGTCATGGCAATTGCACAAAatgggctgcaccagagtgcacactgacctcagaaggatggctgtgtgtgtacatatctgtgtacacttggggtagacagagcatgagaaatgGCCTCAACACCTCCCAAGCACAGAGTGTCCCTGTCATCAACAGTGTGGCC |
| Identified by whole gene mapping | >HNF1B_i6_ins169 | CATCATGACACCCCTCTCTGGAGTCATGGCAATTGCACAAAcatacttcccaagcaagaaccagtgcccagaaacagaaacagatgaaaaggtcaagacttacccactgagggagctggaatgacatcagaggctctcaatcctggaattgaggcacatcaggatcacagactgcattgaaaataagattcctgggccccacctctgaagGCCTCAACACCTCCCAAGCACAGAGTGTCCCTGTCATCAACAGTGTGGCC |

**Supplementary table 2.** Fasta sequences used for final mapping of NGS output of all cDNA pools. Fasta sequences are divided into two categories according to the origin of respective sequences. “Hypothesized” sequences were manually constructed to cover all possible exon deletions. “Identified by whole gene mapping” sequences were identified as a result of reads mapping to the whole HNF1B gene.

**Supplementary table 3**

| **Variant name** | **HGVS description** | **Functional annotation** | **Splicing biotype** | **EEC pool** | **Colorectal carcinoma pool** | **Healthy colon** | **Kidney carcinoma pool** | **Healthy kidney** | **Kidney oncocytoma** | **Pancreatic carcinoma** | **Healthy pancreas** | **Prostate carcinoma** | **Paired healthy tissue** | **Prostate hyperplasia** | **No. of pools containing ASV** | **Known (1) Novel (0)** |
| --- | --- | --- | --- | --- | --- | --- | --- | --- | --- | --- | --- | --- | --- | --- | --- | --- |
| **Number of total reads in each pool (NextSeq kit v2 - 150 cycles)** | | | | **266**  **609** | **427**  **626** | **406**  **352** | **416**  **395** | **387**  **065** | **367**  **393** | **425**  **119** | **406**  **781** | **472**  **820** | **446**  **522** | **434**  **928** |  |  |
| Δ2 | c.345_544del  200 | FS | CΔ | 68 | 0 | 591 | 77 | 277 | 108 | 137 | 226 | 0 | 0 | 210 | 8 | 0 |
| Δ2,3p | c.345_622del  278 | FS | CΔ + SDSΔ | 0 | 61 | 0 | 49 | 160 | 68 | 27 | 0 | 0 | 59 | 182 | 7 | 0 |
| Δ2_4 | c.345_1045del701 | FS | mCΔ | 18 | 0 | 23 | 181 | 61 | 90 | 61 | 37 | 0 | 0 | 0 | 7 | 0 |
| Δ2_5 | c.345_1206del862 | FS | mCΔ | 0 | 0 | 0 | 13 | 5 | 0 | 0 | 0 | 0 | 0 | 0 | 2 | 0 |
| Δ2_6 | c.345_1339del995 | FS | mCΔ | 0 | 0 | 0 | 298 | 0 | 0 | 0 | 0 | 0 | 0 | 813 | 3 | 0 |
| Δ2_7,8p | c.345_1584del1204 | FS | mCΔ + SASΔ | 0 | 0 | 0 | 0 | 43 | 0 | 0 | 0 | 0 | 0 | 0 | 1 | 0 |
| Δ2_8 | c.345_1653del1309 | FS | mCΔ | 0 | 0 | 92 | 368 | 310 | 115 | 184 | 112 | 199 | 0 | 0 | 7 | 0 |
| **3p** | **c.545_622del**  **78** | **IF** | **SASΔ** | 23 750 | 71 077 | 75 375 | 69 140 | 66 016 | 54 341 | 77 678 | 66 348 | 101 630 | 86 642 | 129 509 | **11** | **1** |
| Δ3_4 | c.545_1045del501 | IF | mCΔ | 5 | 59 | 36 | 77 | 87 | 31 | 129 | 90 | 0 | 0 | 0 | 8 | 0 |
| Δ3_5 | c.545_1206del662 | FS | mCΔ | 0 | 0 | 0 | 0 | 0 | 2 | 0 | 0 | 0 | 0 | 0 | 1 | 0 |
| Δ3_5,6p | c.545_1328del784 | FS | mCΔ + SASΔ | 0 | 0 | 0 | 51 | 0 | 0 | 0 | 0 | 0 | 0 | 0 | 1 | 0 |
| Δ3_8 | c.545_1653del1109 | FS* | mCΔ | 0 | 0 | 0 | 0 | 51 | 0 | 0 | 0 | 0 | 0 | 0 | 1 | 0 |
| Δ4_5,3q,6p | c.796_1222del427 | FS | mCΔ + SDSΔ + SASΔ | 0 | 0 | 0 | 0 | 8 | 0 | 0 | 0 | 0 | 0 | 0 | 1 | 0 |
| Δ4,3q | c.799_1045del247 | FS | CΔ + SDSΔ | 0 | 0 | 0 | 0 | 0 | 0 | 6 | 0 | 0 | 0 | 0 | 1 | 0 |
| Δ4_6,3q,7p | c.804_1359del556 | FS | mCΔ + SDSΔ + SASΔ | 2 | 0 | 0 | 0 | 0 | 0 | 0 | 0 | 0 | 0 | 0 | 1 | 0 |
| Δ4 | c.810_1045del236 | FS | CΔ | 0 | 0 | 16 | 16 | 20 | 0 | 124 | 47 | 0 | 0 | 0 | 5 | 0 |
| Δ5_7,4q,8p | c.1039_1548  del510 | IF | mCΔ + SDSΔ + SASΔ | 0 | 0 | 24 | 0 | 0 | 0 | 0 | 0 | 0 | 0 | 0 | 1 | 0 |
| Δ5_6,4q,7p | c.1041_1520  del480 | FS | mCΔ + SDSΔ + SASΔ | 0 | 0 | 211 | 0 | 0 | 0 | 0 | 0 | 0 | 0 | 0 | 1 | 0 |
| Δ5 | c.1046_1206  del161 | FS | CΔ | 36 | 33 | 110 | 261 | 134 | 78 | 376 | 167 | 283 | 302 | 159 | 11 | 0 |
| Δ5,6p | c.1046_1222  del177 | IF | CΔ + SASΔ | 0 | 3 | 0 | 7 | 0 | 19 | 0 | 0 | 0 | 0 | 2 | 4 | 0 |
| Δ5,6p | c.1046_1225  del180 | IF | CΔ + SASΔ | 0 | 0 | 0 | 3 | 0 | 0 | 0 | 0 | 0 | 0 | 0 | 2 | 0 |
| Δ5_6 | c.1046_1339  del294 | IF | mCΔ | 0 | 0 | 0 | 61 | 228 | 0 | 0 | 0 | 0 | 0 | 0 | 2 | 0 |
| Δ5_7 | c.1046_1534  del489 | IF | mCΔ | 0 | 0 | 0 | 230 | 0 | 0 | 0 | 0 | 0 | 0 | 0 | 1 | 0 |
| **Δ5_8** | **c.1046_1653**  **del608** | **IF*** | **mCΔ** | 30 | 89 | 352 | 1 060 | 854 | 893 | 334 | 377 | 0 | 177 | 503 | **10** | **0** |
| Δ6_7,5q | c.1199_1534  del336 | IF | mCΔ + SDSΔ | 0 | 0 | 93 | 0 | 2 | 0 | 11 | 0 | 0 | 0 | 0 | 3 | 0 |
| Δ6 | c.1207_1339  del133 | FS | CΔ | 51 | 27 | 41 | 104 | 57 | 79 | 102 | 126 | 241 | 0 | 0 | 9 | 0 |
| **Variant name** | **HGVS description** | **Functional annotation** | **Splicing biotype** | **EEC pool** | **Colorectal carcinoma pool** | **Healthy colon** | **Kidney carcinoma pool** | **Healthy kidney** | **Kidney oncocytoma** | **Pancreatic carcinoma** | **Healthy pancreas** | **Prostate carcinoma** | **Paired healthy tissue** | **Prostate hyperplasia** | **No. of pools containing ASV** | **Known (1) Novel (0)** |
| Δ6_7 | c.1207_1534  del328 | FS | mCΔ | 0 | 0 | 0 | 211 | 56 | 171 | 112 | 44 | 0 | 0 | 0 | 5 | 0 |
| **Δ6_8** | **c.1207_1653**  **del477** | **IF** | **mCΔ** | 151 | 256 | 259 | 1 365 | 1 159 | 711 | 382 | 840 | 289 | 154 | 1 243 | **11** | **0** |
| Δ7,6q,8p | c.1336_1542  del207 | IF | CΔ + SDSΔ + SASΔ | 18 | 2 | 5 | 3 | 9 | 25 | 14 | 10 | 12 | 20 | 8 | **11** | 0 |
| **Δ7** | **c.1340_1534**  **del195** | **IF** | **CΔ** | 3 480 | 11 051 | 10 324 | 12 076 | 10 737 | 11 514 | 14 830 | 11 984 | 13 961 | 10 910 | 18 297 | **11** | **0** |
| **Δ7_8** | **c.1340_1653**  **del314** | **IF*** | **mCΔ** | 2 277 | 17 034 | 15 496 | 26 969 | 28 172 | 35 372 | 30 741 | 18 500 | 10 801 | 11 725 | 21 414 | **11** | **1** |
| **Δ8** | **c.1535_1653**  **del119** | **IF*** | **CΔ** | 373 | 1 927 | 2 336 | 2 359 | 2 359 | 2 089 | 1 463 | 1 501 | 389 | 745 | 519 | **11** | **0** |
| **Number of total reads in each pool (MiSeq kit v2 - 300 cycles)** | | | | **296 236** | **945 478** | **1 175 448** | **731 641** | **692 952** | **1 119 334** | **1 244 013** | **1 073 030** | **1 268 070** | **1 332 268** | **1 186 521** |  |  |
| ▼106bp_i2 * | c.545-899_549-793ins106 | FS | C▼ | 19 | 0 | 0 | 0 | 0 | 19 | 0 | 5 | 0 | 0 | 0 | 3 | 0 |
| ▼153bp_i4 * | c.1046-10281_1046-10129ins153 | IF, PTC | C▼ | 2 | 7 | 7 | 5 | 5 | 0 | 9 | 20 | 8 | 2 | 0 | 9 | 0 |
| ▼157bp_i4 | c.1046-10281_1046-10125ins157 | FS | C▼ | 9 | 25 | 19 | 21 | 12 | 4 | 32 | 26 | 11 | 15 | 7 | 11 | 0 |
| ▼91bp_i4 * | c.1046-10219_1046-10129ins91 | FS | C▼ | 0 | 0 | 0 | 3 | 0 | 0 | 0 | 0 | 0 | 0 | 0 | 1 | 0 |
| ▼94bp_i4 * | c.1046-10219_1046-10125ins94 | FS | C▼ | 0 | 0 | 5 | 0 | 0 | 0 | 2 | 0 | 0 | 0 | 0 | 2 | 0 |
| ▼91bp_i4 | c.1046-1421_1046-1331ins91 | FS | C▼ | 275 | 204 | 240 | 437 | 320 | 199 | 388 | 494 | 299 | 531 | 298 | 11 | 0 |
| ▼128bp_i4 | c.1046-765_1046-638ins128 | FS | C▼ | 29 | 51 | 52 | 74 | 50 | 27 | 55 | 70 | 40 | 115 | 41 | 11 | 0 |
| ▼99bp_i4 * | c.1046-1421_1046-1331ins91 + c.1046-8_1046-1ins8 | IF | C▼+  SAS▼ | 7 | 0 | 0 | 0 | 0 | 0 | 2 | 0 | 18 | 0 | 0 | 3 | 0 |
| ▼79bp_i5 * | c.1206+1417_1206+1495ins79 | FS | C▼ | 0 | 0 | 17 | 0 | 0 | 2 | 0 | 0 | 0 | 0 | 6 | 3 | 0 |
| ▼148bp_i5 | c.1206+1417_1206+1564ins148 | FS | C▼ | 109 | 71 | 48 | 169 | 112 | 43 | 134 | 198 | 104 | 143 | 18 | 11 | 0 |
| ▼195bp_i6,Δ7_8 * | c.1339+521_1339+715ins195 + c.1340_1653del314 | FS | C▼+mCΔ | 0 | 0 | 0 | 38 | 0 | 0 | 0 | 0 | 0 | 0 | 0 | 1 | 0 |
| ▼92bp_i6 * | c.1339+1620_1339+1711ins92 | FS | C▼ | 0 | 0 | 0 | 0 | 0 | 0 | 10 | 0 | 0 | 0 | 0 | 1 | 0 |
| ▼169bp_i6 | c.1340-1781_1340-1613ins169 | FS | C▼ | 10 | 38 | 26 | 80 | 7 | 3 | 72 | 42 | 5 | 7 | 0 | 10 | 0 |

**Supplementary table 3.** List of the identified HNF1B ASVs in eleven different tissue pools and original sequencing read count for each pool prior to normalization. The paired tumour and healthy tissue pools have a green background, the non-paired pools have an orange background. The “predominant” variants are in bold and highlighted in light blue, and the “predominant candidate” variants are in bold and highlighted in light green. Intron exonizations (based on 2x150bp reads data) are highlighted in light orange. Variants identified exclusively by 2x150bp reads data are in red. The list includes the variant´s name, HGVS nomenclature, presumed functional annotation (FS = frameshift; IF – in frame; FS* - frameshift in last exon 9 with alternative STOP codon), splicing event biotype (Δ = deletion; ▼ = insertion; C = cassette/exon; mC = multicasette/multiexon; SDS = splice donor site shift; SAS = splice acceptor site shift).

**Extended data – Supplementary Figure 3.**

**Original photos of agarose gels**

A)


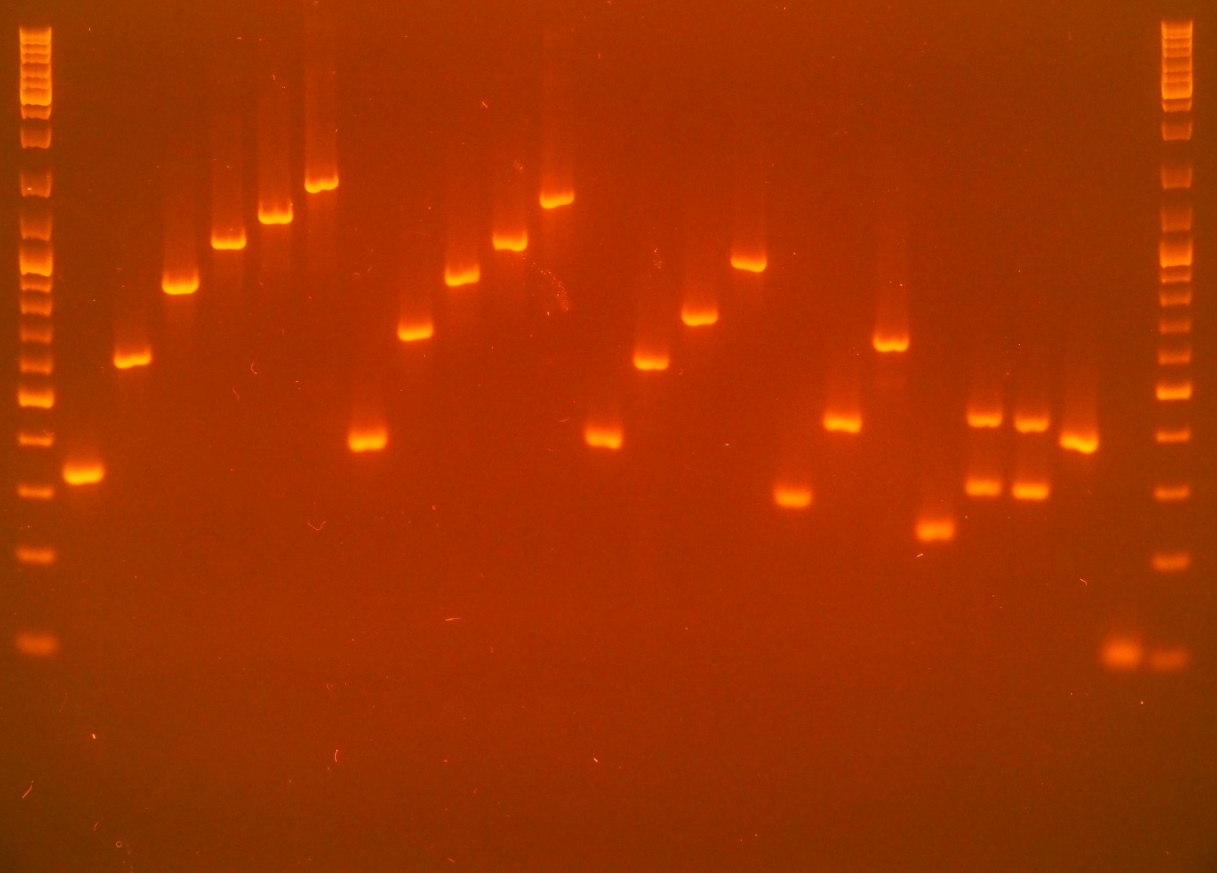


B) C)

**
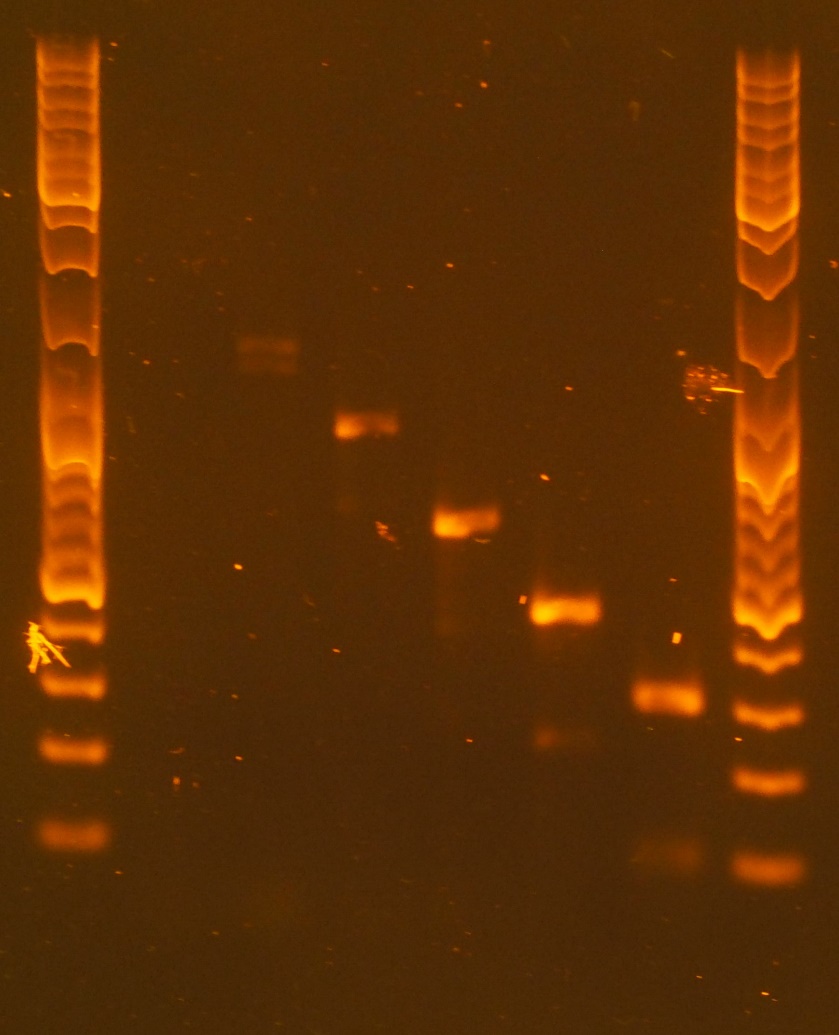
**
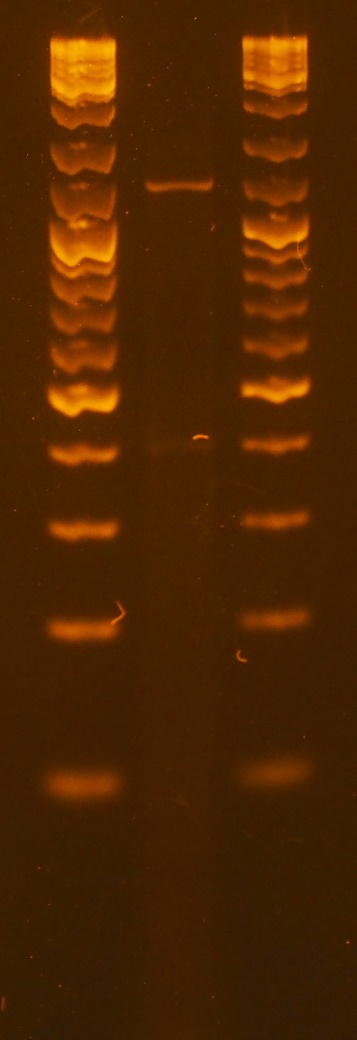

Supplement: Supplementary file 1 — Supplementary Information. [file 41598_2020_63733_MOESM1_ESM.docx]
